# Effects of *Ophiopogon japonicus* oligosaccharides on type 2 diabetes in rats via modulation of gut microbiota and metabolites

**DOI:** 10.3389/fphar.2025.1710883

**Published:** 2025-11-20

**Authors:** Tingyu Yang, Baoting Chen, Jia Fang, Zilin Li, Yiming Liu, Aihua Lin

**Affiliations:** 1 Chinese Medicine Guangdong Laboratory, Guangdong, China; 2 The Second Affiliated Hospital of Guangzhou University of Chinese Medicine, Guangzhou, China; 3 Guangdong Provincial Hospital of Chinese Medicine, Zhuhai, China; 4 Guangdong Provincial Key Laboratory of Clinical Research on Traditional Chinese Medicine Syndrome, Guangzhou, China

**Keywords:** ophiopogon japonicus oligosaccharides, gut microbiota, serum metabolites, short-chain fatty acids, diabetes

## Abstract

**Introduction:**

*Ophiopogon japonicus* oligosaccharides (OJO) is a bioactive component extracted and purified from the traditional Chinese medicinal herb *Radix Ophiopogonis* (Maidong) with significant hypoglycemic effects, although its mechanism of action remains to be further studied.

**Methods:**

This study used a high-fat diet supplemented with streptozotocin to establish a rat model of type 2 diabetes mellitus (T2DM). OJO was administered at low, medium, and high doses for four consecutive weeks. Biochemical indices of glucose and lipid metabolism were measured, and feces, serum, and colonic contents were collected for 16 S rRNA gene sequencing, metabolomics, and gas chromatography–mass spectrometry.

**Results:**

OJO treatment significantly alleviated polyuria and weight loss, ameliorated insulin resistance, and improved glucose and lipid metabolism disorders in T2DM rats. OJO also modulated gut microbiota composition by increasing the *Firmicutes*-to-*Bacteroidota* ratio and regulating key bacterial genera, including decreased *Lactobacillus* and *Prevotella* and increased *unclassified_f_*Lachnospiraceae, *Faecalibaculum*, *norank_f_norank_o_Clostridia_*UCG-014, Christensenellaceae*_R-7_group*, *Romboutsia*, and UCG-005. Additionally, OJO significantly reduced acetic acid and propanoic acid levels. Serum untargeted metabolomic analysis revealed that OJO modulated 40 diabetes-associated metabolites, primarily linked to the synthesis and metabolic pathways of aromatic amino acids and bile acids. Correlation analysis identified significant connections between these metabolic alterations and specific gut microbiota.

**Conclusion:**

OJO exhibits therapeutic potential for T2DM, possibly by regulating gut microbiota and associated metabolites.

## 1 Introduction

Diabetes mellitus has emerged as a significant ‘killer’, severely impacting human health (Majety et al., 2023). The International Diabetes Federation estimated that, as of the end of 2021, there were over half a billion individuals aged 20 to 79 worldwide diagnosed with diabetes (Sun et al., 2022). Type 2 diabetes mellitus (T2DM) is the predominant form of diabetes in adults, constituting around 90% of the total (Zheng et al., 2018). T2DM arises from a combination of hereditary and environmental influences (The, 2017), and its aetiology and pathogenesis are still unclear. Despite the development of many drugs to treat T2DM, a cure has not yet been discovered (Kumar et al., 2021). Moreover, the adverse reactions and poor adherence of existing antidiabetic drugs underscore the urgent need for safer and more efficacious treatment options. Increasing research evidence suggests that plant-derived bioactives exhibit potent hypoglycemic activity coupled with reduced toxicity and minimal adverse effects (Xu et al., 2018; Roy et al., 2019; Zheng et al., 2019; Liu et al., 2022).

*Ophiopogon japonicus* (Thunb.) Ker Gawl., a Liliaceae herbaceous plant, has extensive applications in China, Japan, Vietnam, and many other countries (Chen et al., 2016). Its dried tuberous root, known as *Radix Ophiopogonis*, holds an important place in traditional Chinese medicine and was documented in the *Compendium of Materia Medica* as a remedy for “Xiaoke Bing”, a traditional Chinese medicine term resembling diabetes mellitus (Li, 1,596). Modern pharmacological studies have revealed that *Radix Ophiopogonis* possesses antidiabetic (Ding et al., 2012), immunomodulatory (Lu et al., 2017), anti-inflammatory activities (Kitahiro et al., 2018). In 2024, *Radix Ophiopogonis* was included in China’s List of Substances Traditionally Considered as Both Food and Chinese Medicine, which has facilitated its development as a functional food and dietary supplement (China, 2024). Its bioactive constituents include polysaccharides, saponins, phenolics, and homoisoflavonoids, among which polysaccharides are recognized as the major contributors to its hypoglycemic activity (Wang et al., 2012; Chen et al., 2013; Fang et al., 2018; Zhang et al., 2024). However, polysaccharides have a high molecular weight and complex structures, making in-depth research challenging (Ren et al., 2019). In contrast, oligosaccharides have a lower molecular weight and good water solubility (Liu et al., 2022). They often exhibit stronger biological activity than the corresponding polysaccharides and are considered major active components in many medicinal plants (Bai et al., 2022; Liu et al., 2022). Through systematic research, our team successfully isolated and purified oligosaccharide fractions (500–2,500 Da) from *Radix Ophiopogonis*, achieving a crude yield of 38.52% (Lin et al., 2011; Li et al., 2012; Wu et al., 2017). These fractions demonstrated significant hypoglycemic activity. Specifically, *O. japonicus* oligosaccharides (OJO) significantly elevated hepatic glucokinase activity, reduced phosphoenolpyruvate carboxykinase activity, increased glucagon-like peptide-1 levels, inhibited glucagon secretion, and enhanced insulin activity (Li et al., 2012). Therefore, OJO is one key bioactive component responsible for the hypoglycemic effects of *Radix Ophiopogonis*, and further research into its antidiabetic efficacy and mechanisms is of great significance.

Recent pharmacological studies indicate that dysregulation of gut microbiota has been implicated in T2DM pathogenesis (Qin et al., 2012; Ma et al., 2019). Disruptions in the balance between *Firmicutes* and *Bacteroidota* and alterations in bacteria involved in butyrate production may give rise to T2DM (Larsen et al., 2010; Sato et al., 2014; Sircana et al., 2018; Zhu et al., 2020). Dysbiosis of the gut microbiota results in significant alterations in metabolites, including tryptophan, bile acids (BAs), branched-chain amino acids (BCAAs), and short-chain fatty acids (SCFAs), which are implicated in T2DM pathogenesis and are essential for host-microbe interaction (Du et al., 2022; Wu et al., 2023). Gut microbiota-mediated breakdown of non-digestible carbohydrates (e.g., oligosaccharides, resistant starches, and polysaccharides) serves as the major pathway for SCFA production (Yao et al., 2020). External stimuli may lead to alterations in gut microbiota, subsequently modulating the levels and types of SCFAs (Cao et al., 2023). Therefore, rebalancing the gut microbiota and regulating metabolite production are regarded as novel strategies for preventing T2DM.

Functional oligosaccharides, characterised by their non-digestibility, low-caloric content, and prebiotic properties, represent a potential therapy for diabetes (Zhu et al., 2019). Recently, many functional oligosaccharides are known to alleviate T2DM via the regulation of gut microbiota (Song et al., 2020; Liu et al., 2022), prompting us to examine whether gut microbiota and associated metabolites are linked to the response to OJO treatment in T2DM.

## 2 Materials and methods

### 2.1 Materials

*Radix Ophiopogonis* was purchased from Guangzhou Qingping traditional Chinese medicine market and was identified as root of *O. japonicus (Thunb.) Ker-Gawl.* by Chief Pharmacist Peng Wei from the School of Life Sciences, Sun Yat-sen University. The preparation of OJO was performed as previously reported (Xu et al., 2017). Briefly, *Radix Ophiopogonis* was pretreated with 95% ethanol under reflux for 2 h to remove impurities. The residues were then extracted twice with 10 volumes of distilled water, and the combined extracts were concentrated under reduced pressure. Ethanol was added to a final concentration of 80%, and the mixture was allowed to stand for 12 h to precipitate. The precipitate was sequentially washed with absolute ethanol, anhydrous ether, and acetone, and then lyophilized to obtain the crude extract. The crude oligosaccharide fraction was further purified on a Sephadex G-75 column (3 × 80 cm) and lyophilized to yield purified OJO.

### 2.2 Characterization of OJO

The sulfuric acid–anthrone assay was employed to measure the oligosaccharide content of OJO. Each measurement was repeated three times, and the mean value was used for analysis.

The molecular weight range of OJO was analyzed by matrix-assisted laser desorption/ionization time-of-flight mass spectrometry (MALDI-TOF-MS). The purified OJO was dissolved in ultrapure water to prepare a 2 μg · mL^-1^ solution. Then, 0.5 μL of the sample solution was mixed with 0.5 μL of matrix solution (5 mg · mL^-1^ 2,5-dihydroxybenzoic acid) for analysis.

The monosaccharide composition of OJO was determined as previously reported using gas chromatography–mass spectrometry (GC-MS) (Lin et al., 2011). Briefly, the oligosaccharides were hydrolyzed with 2 M trifluoroacetic acid at 120 °C, and the excess acid was removed by repeated co-distillation with methanol. The hydrolysates were then reduced with sodium borohydride and treated with glacial acetic acid to decompose the excess reagent. After evaporation to dryness to remove borate, the reduced products were acetylated with acetic anhydride in pyridine before further analysis.

### 2.3 Animals

Seventy male Sprague-Dawley rats were purchased from the Laboratory Animal Centre of Southern Medical University (licence number SCXK (Guangdong) 2016–0041) and randomly assigned to the control group (CON, n = 10), which received a basal diet, or the high-fat diet group (HFD, n = 60), which was maintained on a high-fat diet. Details of the diet composition are provided in the Supplementary Material. The rats underwent a 12-h fast after completing a 4-week feeding period. The HFD group received 30 mg · kg^-1^ of streptozotocin (STZ), with the CON group administered saline of equal volume. Fasting blood glucose (FBG) was assessed at 72 h with the ACCU-CHEK Performa glucometer (Roche diagnostics, Mannheim, Germany) and retested 1 week later. Successful modelling was confirmed if FBG remained consistently above 16.7 mmol · L^-1^. Rats that did not meet the modelling criteria were administered a suitable STZ dose, and FBG measurements were continued. Ultimately, the T2DM model was successfully developed in fifty rats, which were then assigned at random to five groups of ten: the diabetes model group (MOD), the metformin group (MET), and the OJO treatment groups: low, medium, and high-dose (L-OJO, M-OJO, and H-OJO). In the MET group, each rat was administered 250 mg · kg^-1^ daily of metformin hydrochloride tablets (Sino-American Shanghai Squibb Pharmaceuticals Ltd., Shanghai, China). In the OJO intervention groups, 112, 225, and 450 mg · kg^-1^ doses were administered. Saline in the same volume was administered to both the CON and MOD groups. All groups underwent gavage treatment for 4 weeks, with daily body weight measurements to adjust the administered volume. FBG was measured weekly with a glucometer after 12 h of fasting. At the end of the intervention, oral glucose tolerance tests (OGTT) were conducted. Thereafter, rats were housed in metabolic cages for 24 h for urine collection. Blood was then extracted via the abdominal aorta following anesthesia of all rats with 10% chloral hydrate. Serum, faeces, and colon contents were collected and stored for subsequent testing. Experiments involving animals complied with the standards set by the Experimental Animal Centre of Guangdong Provincial Hospital of Traditional Chinese Medicine and received approval from the Animal Ethics Committee of Guangdong Provincial Hospital of Traditional Chinese Medicine (Reference number 44002100024609).

### 2.4 Biochemical analysis

Serum samples frozen at −80 °C were removed and re-dissolved at 4 °C. Total cholesterol (TC) and triglycerides (TG) were determined by assay kits, E1005 and E1003 (Applygen Technologies Inc., Beijing, China). High-density lipoprotein cholesterol (HDL-C) and low-density lipoprotein cholesterol (LDL-C) were measured according to commercial kits purchased from Nanjing Jiancheng Bioengineering Institute (Nanjing, China). Fasting insulin (FINS) was determined as described previously, and the homeostatic model assessment of insulin resistance (HOMA-IR) was calculated accordingly (Chen et al., 2024). FBG, OGTT, FINS, and HOMA-IR data for this model have been reported previously (Chen et al., 2024).

### 2.5 Gut microbiota analysis

Microbiological analysis was conducted by Majorbio Bio-Pharm Technology Co., Ltd. (Shanghai, China). DNA was isolated from faecal samples and subsequently applied to amplify the 16S rRNA V3–V4 region for sequencing. These amplicons were detected through 2% agarose gel. After passing the test, the amplicons were purified and assayed for quantification, followed by construction of Miseq libraries and on-board sequencing.

Raw sequencing data underwent quality control and splicing with the Trimmomatic and FLASH software. Using a 97% similarity criterion, non-redundant sequences were grouped into operational taxonomic units (OTUs), and representative sequences were annotated to identify species, followed by alpha and beta diversity analysis. Graphical and statistical analysis were conducted on the Majorbio Cloud, with *P* < 0.05 denoting statistical significance.

### 2.6 Serum untargeted metabolomics analysis

The analytical method for serum samples is provided in the spplementary information.

Compound Discoverer 3.1 software served to process the raw UHPLC-MS/MS data, producing a data matrix that contained the grouping information, mass-to-charge ratio, normalised peak areas, and retention time. Subsequently, SIMCA-P 14.1 software was used to import the data matrix for statistical analysis, including Principal Component Analysis (PCA) and Orthogonal Partial Least Squares Discriminant Analysis (OPLS-DA). Significantly different metabolites, with *P* < 0.05 and Variable Importance in Projection (VIP) > 1.5, were classified as different and further identified. Fitted molecular formulas and secondary fragmentation spectra for the corresponding mass-to-charge ratios were obtained using Compound Discoverer 3.1 software. The Metlin, Mass Bank, and Human Metabolome Database databases were searched, and the secondary spectra were compared. Compound structures were determined based on MS/MS fragment ion information and established rules of chemical bond cleavage. The differential metabolites were subjected to pathway enrichment analysis using MetaboAnalyst 5.0 (https://www.metaboanalyst.ca/). Metabolic pathways linked to the differential metabolites were analysed using the KEGG database.

### 2.7 SCFAs analysis

Determination of SCFAs in colonic contents by GC-MS. 0.1 g of colonic content was added to 1 mL of water containing two-ethylbutanoic acid as the internal standard and 0.5% phosphoric acid, followed by thorough grinding. The mixture was sonicated for 30 min on ice, then left to stand and centrifuged for 15 min. The supernatant was separated, then 500 μL of ethyl acetate was added for extraction and vortexed, followed by ultrasound and centrifugation. Then collected supernatant was analysed using GC-MS. Injection volume was 1 μL with a split ratio of 10:1 and a flow rate of 1.0 mL min^-1^. The injection port temperature was set to 260 °C. The initial column temperature was 80 °C, ramped at 40 °C · min^-1^–120 °C, then at 10 °C · min^-1^–200 °C, and finally holding at 230 °C for 3 min. The quadrupole, EI ion source, and transfer line were programmed to 150 °C, 230 °C, and 230 °C, respectively. Selected ion monitoring was performed with an electron energy of 70 eV.

### 2.8 Statistics

Mean ± standard deviation was employed to express the results. Data analysis and graph generation were conducted using SPSS 23.00 and GraphPad Prism 8.3, with independent sample t-tests and one-way analysis of variance used for statistical evaluation. Results were considered statistically significant if *P* < 0.05.

## 3 Results

### 3.1 Characterization of OJO

The total oligosaccharide content of the crude extract, as determined by the sulfuric acid–anthrone method, was 86.73%, and after gel filtration purification, it increased to 97.64%. MALDI-TOF-MS analysis revealed that the molecular masses of OJO ranged from 500 to 2,500 Da (Supplementary Figure S1). GC-MS analysis indicated that OJO was composed of glucose, fructose, and galactose (Lin et al., 2011).

### 3.2 OJO improves glucose and lipid metabolism in T2DM rats

**FIGURE 1:**
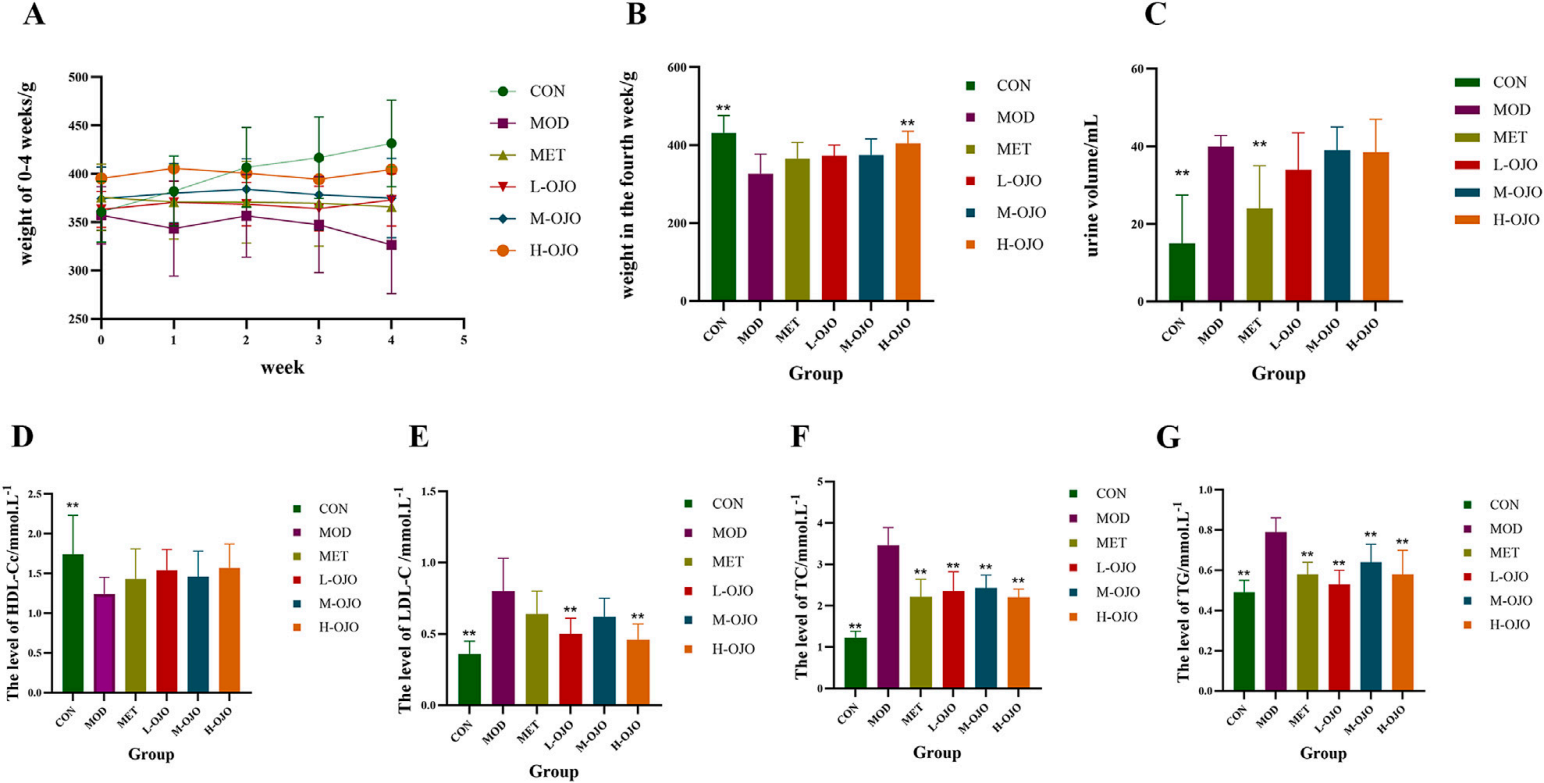
Influence of OJO on body weight, urine volume, and serum lipid levels in T2DM rats. **(A)** Change in body weight from 0 to 4 weeks of administration **(B)** body weight of each group at week 4; **(C)** urine volume **(D)** HDL-C; **(E)** LDL-C; **(F)** TC; **(G)** TG. ^#^ *P* < 0.05, ^##^ *P* < 0.01 vs. M-OJO; ^*^ *P* < 0.05, ^**^ *P* < 0.01 vs. MOD.

Before STZ injection, body weight in the HFD group increased rapidly. However, after STZ administration for model induction, the body weight began to decrease significantly. The changes in body weight during 0–4 weeks of drug administration are illustrated in Figure 1A. Throughout drug intervention, the CON group rats exhibited sustained and stable weight gain compared to the MOD group, and rats in the other treatment groups also showed a trend of slow weight increase. After 4 weeks of treatment, no significant differences in body weight were observed among the L, M, and H-OJO groups (Figure 1B). However, rats from the H-OJO group exhibited a significantly higher body weight than those in the MOD group (*P* < 0.01). OJO treatment notably alleviated the weight loss observed in diabetic rats. Polyuria was also reduced, and the volume in the MET group was markedly smaller than in the MOD group (*P* < 0.01). The OJO group also exhibited reduced urine volume, but the difference between the OJO and MOD groups was not statistically significant, and the results are shown in Figure 1C. The results of FBG, OGTT, FINS, and HOMA-IR have been reported previously (Chen et al., 2024). In short, OJO treatment improved glucose metabolism in T2DM rats, reflected by reduced FBG, FINS, HOMA-IR, and improved glucose tolerance, with the H-OJO group exhibiting the strongest effect.

Next, we evaluated how OJO affected lipid profiles. As illustrated in Figures 1D–G, rats in the MOD group had significantly higher serum levels of TC, TG, and LDL-C compared to the CON group (*P* < 0.01), while serum HDL-C levels were notably reduced (*P* < 0.05). Abnormal lipid profiles in rats from all drug intervention groups showed improvement after 4 weeks of treatment, with the intervention effects of OJO as effective as the MET group. In contrast to the MOD group, HDL-C levels were increased in all drug intervention groups, but none of the differences were statistically significant, while LDL-C levels were decreased, with a significant reduction observed in the H-OJO and L-OJO groups (*P* < 0.01). All drug intervention groups significantly reduced levels of TC and TG in T2DM rats (*P* < 0.01).

### 3.3 OJO regulates the gut microbiota in T2DM rats

**FIGURE 2:**
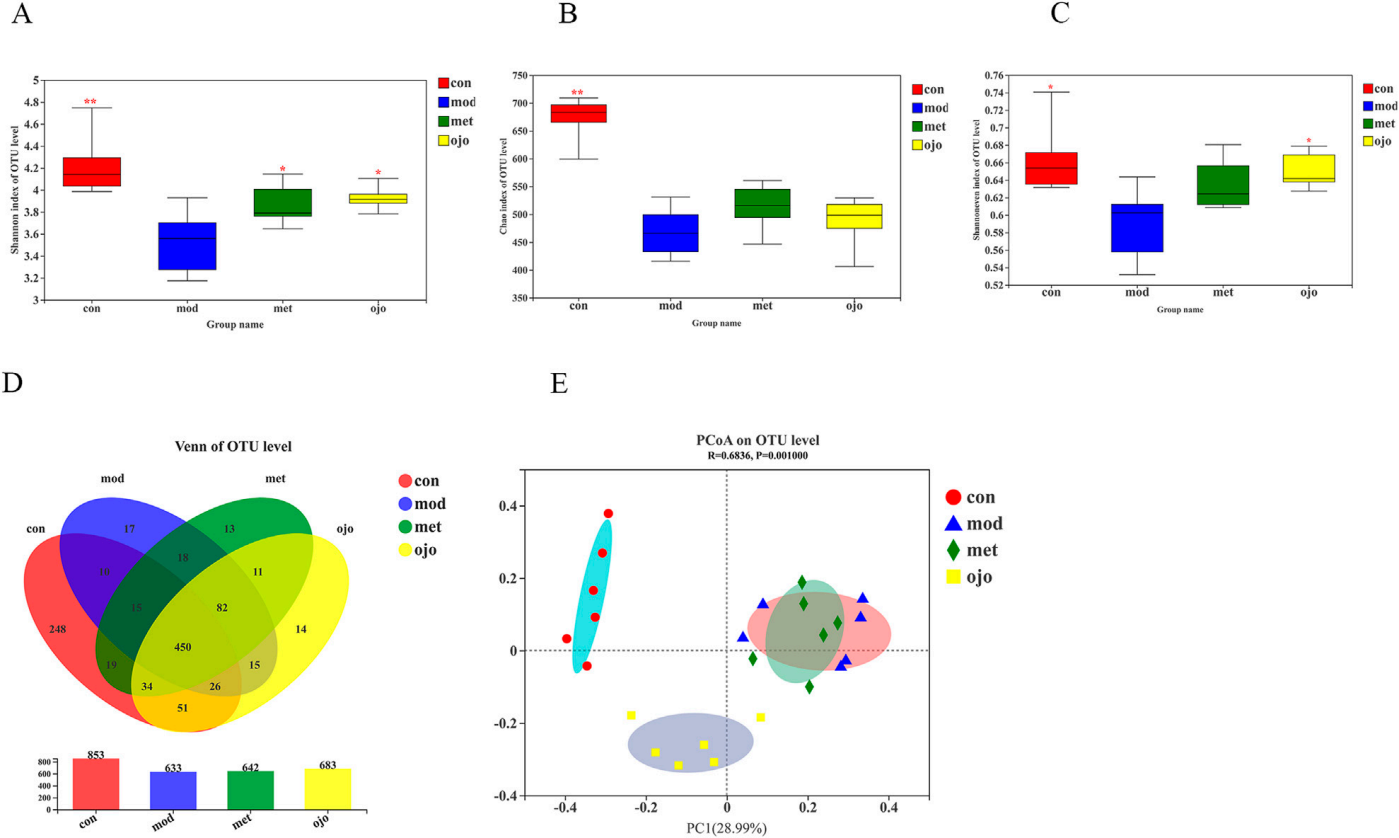
Analysis of gut microbiota α-diversity and β-diversity. **(A)** Shannon diversity index; **(B)** Chao richness index; **(C)** Shannon evenness index; **(D)** Venn of OTU level; **(E)** PCoA analysis. ^*^ *P* < 0.05, ^**^ *P* < 0.01 vs. MOD.

Figures 2A,B demonstrate that the MOD group exhibited a significant reduction in both Shannon index (representing species diversity) and Chao index (representing species richness) in comparison with the CON group (*P* < 0.01). Meanwhile, the Chao index in the drug intervention group was increased but not statistically significant in comparison with the MOD group, while the Shannon index in the drug intervention group was markedly higher (*P* < 0.05), suggesting that both OJO and MET increased the diversity of intestinal microorganisms in diabetic rats. Figure 2C shows that in the OJO group, the Shannon evenness index (reflecting species evenness) was also markedly elevated (*P* < 0.05). The reasonableness of the sequencing data was verified using the Rarefaction and Shannon curve, which indirectly reflected the richness of species in the samples. Both curves tended to flatten out, and the coverage was greater than 99.7% (Supplementary Figure S2A, B). As shown in Figure 2D, 501 OTUs were shared between the CON and MOD groups, whereas 561 OTUs were shared between the OJO and CON groups, indicating that OJO treatment tended to normalize the gut microbiota composition in T2DM rats. In contrast, the MET and CON groups shared 518 OTUs, suggesting a less pronounced effect. We analysed β-diversity to better understand the changes in gut microbiota structure and composition induced by OJO. As illustrated in Figure 2E, PCoA analysis revealed a clear separation of gut microbiota from the CON, MOD, OJO, and MET groups into three categories, reflecting significant microbiota changes in diabetic rats and the modulatory potential of OJO. The NMDS analysis results agreed with the PCoA analysis (Supplementary Figure S2C). ANOSIM analysis further confirmed significant differences in the gut microbiota community structures among the groups (*P* = 0.001; Supplementary Figure S2D).

**FIGURE 3:**
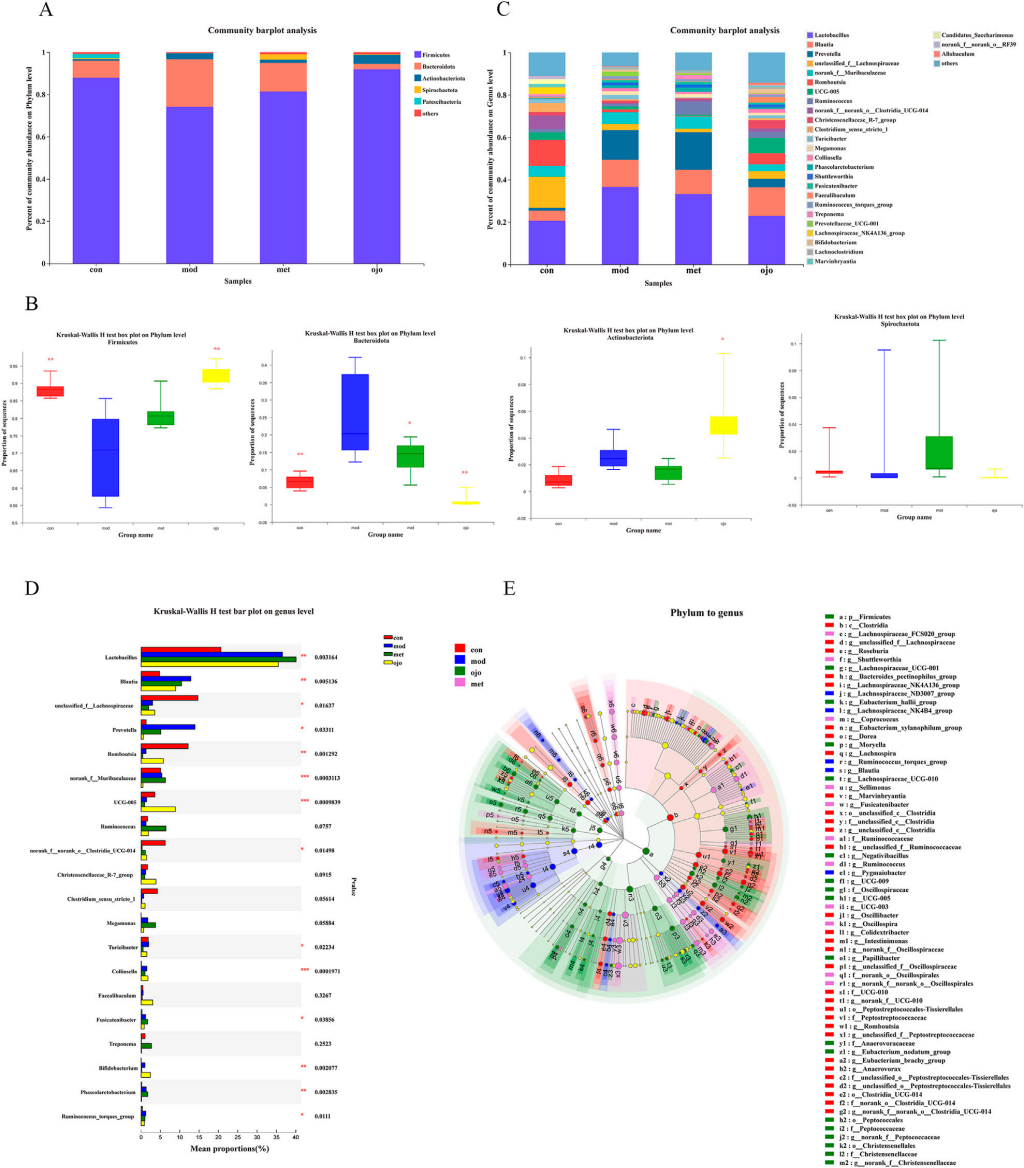
OJO alters the gut microbiota in T2DM rats. **(A)** Bar chart of microbial community composition at the phylum level; **(B)** The relative abundance of *Firmicutes*, *Bacteroidota*, *Actinobacteriota*, *Spirochaetota* **(C)** Bar chart of microbial community composition at the genus level; **(D)** Differential analysis of gut microbiota at the genus level; **(E)** Multi-level species differential analysis from phylum to genus using LefSe. Nodes of different colours represent microbial taxa significantly enriched in specific groups and contributing to intergroup differences, while pale yellow nodes indicate no significant differences across groups. Species abundance is visualized by node size. ^*^ *P* < 0.05, ^**^ *P* < 0.01, ^***^ *P* < 0.001 vs. MOD.

At the phylum level, significant changes in the relative abundance of dominant phyla, such as *Firmicutes*, *Bacteroidota*, and *Actinobacteriota*, were observed (Figures 3A,B). The MOD group exhibited marked reduction in *Firmicutes* abundance (*P* < 0.01) while showing notable rise in *Bacteroidota* (*P* < 0.01) relative to the CON group. OJO treatment led to a notable rise in *Firmicutes* abundance (*P* < 0.01), along with a marked reduction in *Bacteroidota* (*P* < 0.01) in comparison with the MOD group. Additionally, OJO increased the abundance of *Actinobacteriota* (*P* < 0.05). MET also downregulated *Bacteroidota* abundance in diabetic rats (*P* < 0.05).

At the genus level (Figure 3C), the CON group exhibited relatively high abundances of *Lactobacillus* (20.63%), *unclassified_f_*Lachnospiraceae (14.71%), and *Romboutsia* (12.19%). In the MOD group, *Lactobacillus* was increased (36.58%), and both *Blautia* (12.88%) and *Prevotella* (13.95%) also showed relatively high abundances. In the OJO group, *Lactobacillus* abundance decreased to a level comparable to that in the CON group (22.91%). In contrast, the dominant genera in the MET group showed no obvious changes compared to the MOD group. Further analysis revealed that 70% of the top 20 genera in abundance changed significantly. Figure 3D; Supplementary Figure S3 show that the abundance of *Lactobacillus*, *Blautia*, and *Prevotella* was markedly elevated (*P* < 0.01 or 0.05), while *unclassified_f_*Lachnospiraceae, *Romboutsia*, Lachnospiraceae*_*NK4A136_group, and *norank_f_norank_o_Clostridia_*UCG-014 were markedly reduced (*P* < 0.01 or 0.05) in the MOD group compared to the CON group. In contrast, after OJO intervention, the abundance of *Lactobacillus* and *Prevotella* decreased without reaching statistical significance compared to the MOD group, and *unclassified_f_*Lachnospiraceae, *Faecalibaculum*, Christensenellaceae*_*R-7_group (*P* < 0.05), *norank_f_norank_o_Clostridia*_UCG-014, *Romboutsia*, and UCG-005 (*P* < 0.05) increased. Overall, it can be inferred that during the OJO intervention, the gut microbiota structure in T2DM rats exhibited changes at both the phylum and genus levels, showing a trend toward normalization.

Differential bacteria in each group were compared using linear discriminative analysis (LDA) effect size (LEfSe). As shown in Figure 3E (LDA threshold of 2), there were significant differences in the microbiota distribution from phylum to genus levels among the groups. The CON group was significantly enriched with *Patescibacteria*, *Clostridia*, Peptostreptococcaceae, and *Romboutsia*. In contrast, the MOD group had fewer significantly enriched communities, mainly including *Bacteroidota*, *Prevotella*, and *Blautia*. OJO significantly regulated the dysbiosis of gut microbiota in diabetic rats, markedly increasing the abundance of communities, including *Firmicutes* and *Actinobacteriota* and their subcategories. The MET group had significantly enriched *Spirochaetota* and *Lactobacillus*.

### 3.4 Effects of OJO on SCFAs and correlation analysis

**FIGURE 4:**
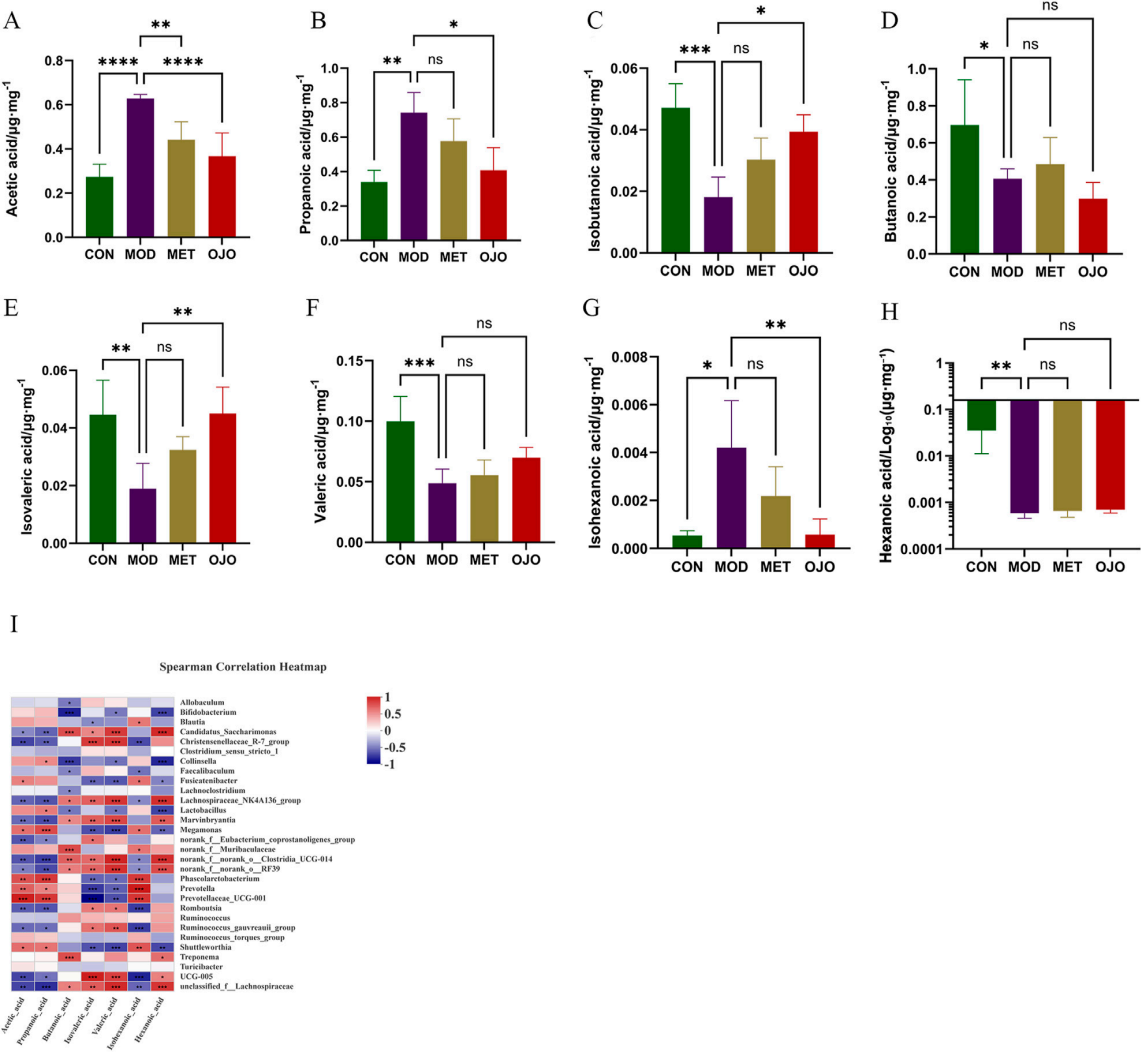
Changes in SCFAs in the colonic content samples of T2DM rats treated with OJO. Due to the substantial difference between the data of other groups and the CON group, the y-axis in Figure 4H is presented on a logarithmic (log10) scale. **(A)** acetic acid; **(B)** Propanoic acid; **(C)** Isobutanoic acid; **(D)** Butanoic acid; **(E)** Isovaleric acid; **(F)** Valeric acid; **(G)** Isohexanoic acid; **(H)** Hexanoic acid; **(I)** Spearman correlation of gut microbiota with SCFAs. ^*^ *P* < 0.05, ^**^ *P* < 0.01, ^***^ *P* < 0.001, ^****^ *P* < 0.0001 vs. MOD.

Figures 4A–H shows the results of our targeted analysis of eight SCFAs in rat colonic contents. Compared with the CON group, the MOD group showed significantly increased levels of acetic acid, propanoic acid and isohexanoic acid. In contrast, the levels of butanoic acid, valeric acid, hexanoic acid, isobutanoic acid and isovaleric acid were significantly reduced. The OJO group showed markedly reduced acetic acid and propanoic acid levels relative to the MOD group (*P* < 0.0001 and *P* < 0.05, respectively), and the MET group also exhibited a marked reduction in acetic acid levels (*P* < 0.01). Additionally, diabetic rats treated with OJO exhibited significantly increased levels of isobutanoic acid, isovaleric acid, and valeric acid, whereas the effect of MET on these SCFAs was insignificant.

The relationship between SCFAs and gut microbiota was evaluated using Spearman’s correlation analysis, as shown in Figure 4I, revealing significant correlations between the seven SCFAs and most genera.

### 3.5 Metabolomics analysis of serum samples

**FIGURE 5:**
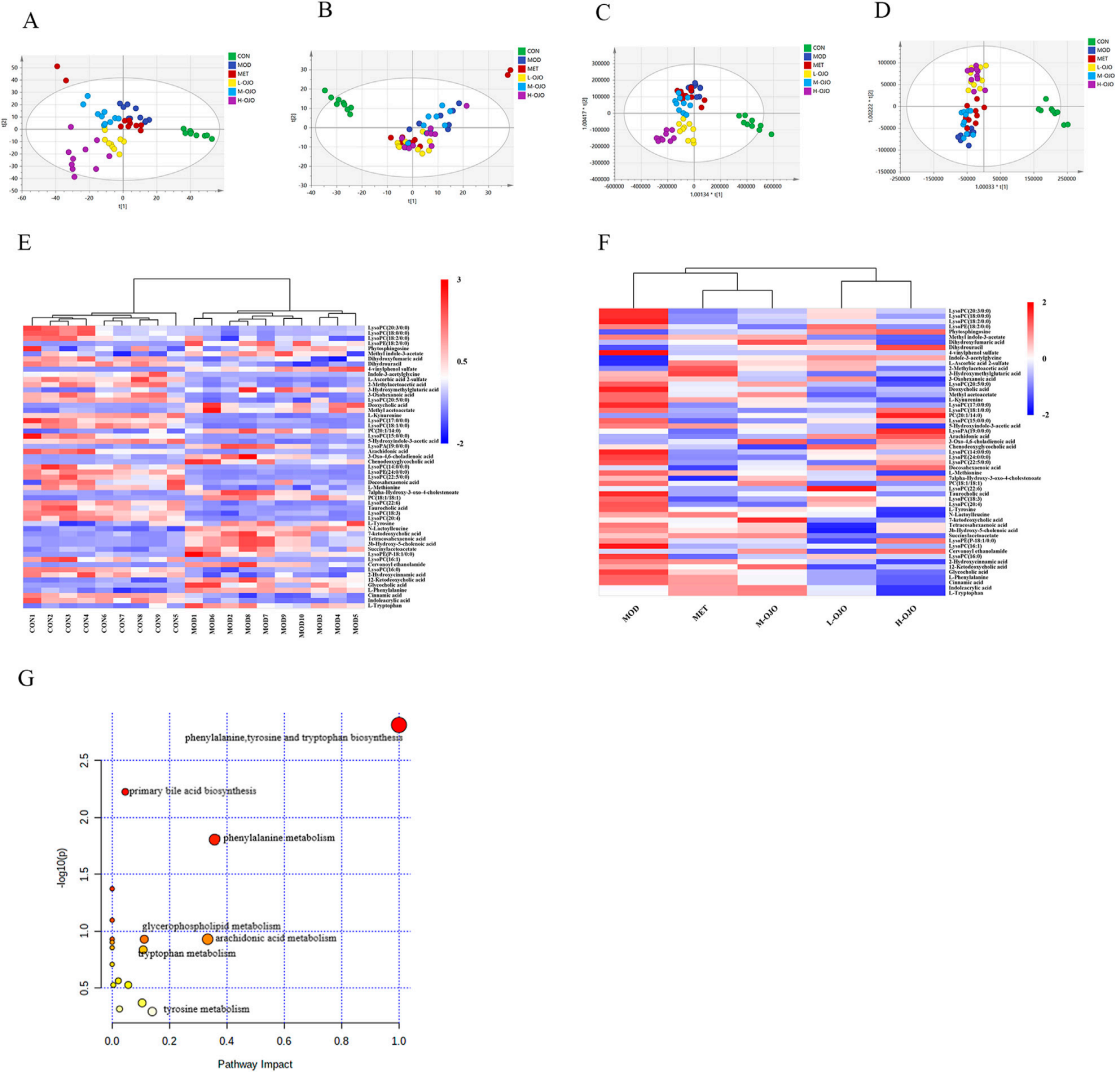
OJO treatment induced notable changes in serum metabolites in T2DM rats. Scatter plot of PCA scores in **(A)** the positive and **(B)** the negative ion mode. OPLA-DA score chart in **(C)** the positive and **(D)** the negative ion mode. Heat maps showed the expression levels of significantly differential metabolites: **(E)** CON vs. MOD, and **(F)** MOD vs. drug treatment groups, where higher concentrations appear with stronger red tones and lower concentrations with deeper blue tones. **(G)** KEGG pathway mapping revealed metabolic alterations linked to differential metabolites.

To investigate metabolite differences among normal, T2DM, and OJO intervention rats, an untargeted metabolomics approach was employed using serum samples. Chromatographic peaks were extracted and identified using Compound Discoverer 3.1 software, resulting in 1,190 negative and 3,334 positive ion peaks. The PCA score plot revealed distinct separation in the serum metabolic profiles among the different groups of rats (Figures 5A,B).

To better demonstrate the group differences, we utilized a supervised OPLS-DA model. In Figures 5C,D, all drug treatment groups are distinctly separated from the MOD group. In particular, the positions of the L-OJO and H-OJO groups were further from the MOD group and closer to the CON group, suggesting that the serum metabolism of T2DM rats after OJO intervention tends to return to normal levels. *R* ^*2*^ and *Q* ^*2*^ are used to illustrate the fitting and predictive capabilities of the model. In Figure 5C, *R* ^*2*^ *X* = 0.786, *R* ^*2*^ *Y* = 0.804, *Q* ^*2*^ = 0.629, and in Figure 5D, *R* ^*2*^ *X* = 0.812, *R* ^*2*^ *Y* = 0.691, *Q* ^*2*^ = 0.418. These values collectively indicate that the results are stable and predictable. To validate the reliability of the model, a permutation test (200 times) was conducted, as shown in Supplementary Figure S4A,B. All *R* ^*2*^ and *Q* ^*2*^ values were lower than the original values, and the intercepts of the blue *Q* ^*2*^ regression lines are negative, indicating the effectiveness of this OPLS-DA model fitting.

Next, we compared the CON group with the MOD group and each drug treatment group with the MOD group to identify differential metabolites, ultimately identifying 55 metabolites associated with diabetes (27 downregulated and 28 upregulated, Supplementary Table S1). Figure 5E also revealed significant changes in serum metabolites of diabetic rats, with significant increases in BAs, phosphatidylcholine, amino acids, and ketone bodies, and significant decreases in lysophosphatidylcholine, indoles, and keto acids. However, following OJO intervention, this trend was reversed, with 40 metabolites showing alterations, notably decreased BAs and amino acids and increased lysophosphatidylcholine, certain indole derivatives, and cinnamic acid-related metabolites (Figure 5F). This indicates that OJO supplementation reversed the serum metabolite dysregulation induced by diabetes. Specifically, primary BAs (taurocholic acid, glycocholic acid, and chenodeoxyglycocholic acid) and secondary BAs (deoxycholic acid, 12-ketodeoxycholic acid, and 7-ketodeoxycholic acid) were significantly increased in T2DM rats. However, after OJO treatment, the levels of 12α-hydroxylated (12αOH) BAs, including taurocholic acid, glycocholic acid, deoxycholic acid, and 7-ketodeoxycholic acid were reduced, while the levels of non-12α-hydroxylated (non-12αOH) BAs like chenodeoxyglycocholic acid increased. Furthermore, OJO can reduce the elevated levels of L-phenylalanine and L-tyrosine in diabetic rats while increasing the diminished levels of cinnamic acid and 2-hydroxycinnamic acid. Eighteen metabolic pathways were identified through pathway enrichment of the differential metabolites, including phenylalanine, tyrosine, and tryptophan biosynthesis, primary bile acid biosynthesis, and phenylalanine metabolism (Figure 5G).

### 3.6 Exploring the correlation of gut microbiota with physicochemical indicators and BAs

**FIGURE 6:**
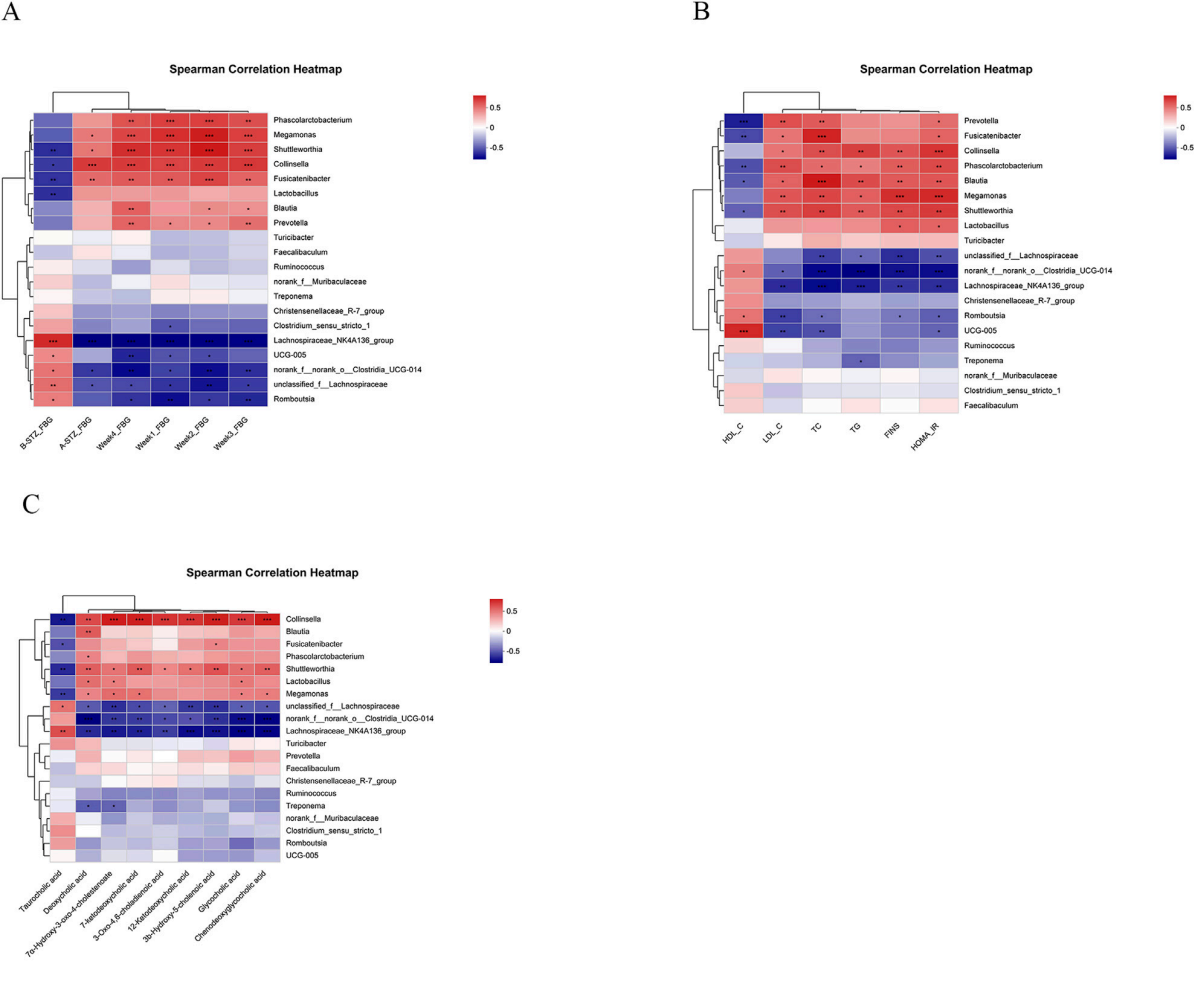
Results of Spearman correlation analysis. **(A)** Correlation analysis of FBG with the 20 most abundant genera. B-STZ_FBG: FBG before STZ treatment; A-STZ_FBG: FBG after STZ treatment; Week1_FBG: FBG in the first week; Week2_FBG: FBG in the second week; Week3_FBG: FBG in the third week; Week4_FBG: FBG in the fourth week. **(B)** Correlation analysis physicochemical indicators with the 20 most abundant genera. **(C)** Correlation analysis BAs with the 20 most abundant genera. ^*^ *P* < 0.05, ^**^ *P* < 0.01, ^***^ *P* < 0.001.

To analyze the association between gut microbiota and physicochemical indicators and the major differential metabolite BAs, we performed Spearman correlation analyses on the top 20 genera in abundance (Figures 6A–C), which showed that *norank_f_norank_o_Clostridia_*UCG-014 and *unclassified_f_*Lachnospiraceae were negatively correlated with diabetes-related physicochemical indicators as well as BAs (*p* < 0.05 or 0.01).

## 4 Discussion

Given the multifactorial nature of diabetes, current treatments focus on controlling blood glucose levels and preventing complications rather than achieving a permanent cure (The Lancet 2017). However, conventional antidiabetic medications are often associated with side effects, including hypoglycemia, gastrointestinal discomfort, and weight gain, and plant bioactives may offer a more suitable approach for long-term diabetes management. Functional oligosaccharides that resist digestion and absorption have recently gained attention as a promising area of research in diabetes treatment (Zhu et al., 2019). Various oligosaccharides from dual-purpose food–medicine plants, such as Lycium barbarum oligosaccharides (Liu et al., 2022), Rehmannia glutinosa oligosaccharides (Li et al., 2020), and Codonopsis pilosula oligosaccharides (Bai et al., 2022), have been reported to ameliorate T2DM, with their hypoglycaemic effects primarily attributed to their role in modulating the gut microbiota. Our research group was the first to extract and purify OJO from *Radix Ophiopogonis*, a classical medicinal food homologous plant, with OJO exhibiting significant hypoglycaemic effects against T2DM (Lin et al., 2011; Li et al., 2012). This study is the first to demonstrate that OJO function as a prebiotic, treating glucolipid metabolism disorders through modulation of gut microbiota and improvement of microbial metabolites.

The HFD combined with the STZ-induced T2DM rat model is commonly used to assess the efficacy of drugs in treating T2DM (Srinivasan et al., 2005; Chao et al., 2018). FBG, OGTT, HOMA-IR, body weight, urine volume, and lipid profile (including TG, TC, LDL-C, and HDL-C) were the main parameters used to assess the severity of T2DM (Kim et al., 2023). In this study, diabetic rats exhibited marked hyperglycaemia, dyslipidaemia, and insulin resistance. Following OJO intervention, significant reductions in FBG, lipid levels, and HOMA-IR were observed (*P* < 0.01), indicating an improvement in glucose and lipid metabolism disorders. Furthermore, OJO enhanced glucose tolerance in diabetic rats. STZ-induced diabetic rats displayed typical symptoms of weight loss, polyuria, polyphagia, and polydipsia (Araiza-Saldana et al., 2015; Miaffo et al., 2021), and our findings show that OJO alleviated these clinical symptoms. These results showed the significant efficacy of OJO on T2DM.

Increasing studies indicate that an imbalance in gut microbiota is strongly linked to various diseases, including T2DM, obesity, cardiovascular disease, and inflammatory bowel disease, among others (Illiano et al., 2020; Zuo et al., 2023). The possible effects that gut microbiota may have on T2DM have been a major focus of research over the past decade (Baars et al., 2024). Recent studies confirm a difference in gut microbiota composition between healthy individuals and T2DM patients, typically characterised by lower microbial diversity and richness (Le Chatelier et al., 2013; Garcia-Gutierrez et al., 2024). This study also revealed a marked reduction in the Shannon and Chao indices in rats from the MOD group, indicating that HDF/STZ-induced T2DM model similarly exhibits decreased microbial diversity and richness. OJO and MET interventions significantly increased the diversity of T2DM rats, with OJO also demonstrating a marked enhancement in species evenness. β-diversity analysis also revealed that the gut microbiota in T2DM rats was notably altered and could be effectively modulated by OJO. In addition, changes in the *Firmicutes*/*Bacteroidota* ratio of the gut microbiota are regarded as key contributors to the onset of T2DM (Pan et al., 2024). Our findings revealed a considerable decline in *Firmicutes* and a marked increase in *Bacteroidota* in T2DM rats. However, OJO intervention reversed this trend and caused a notable increase in the *Firmicutes*/*Bacteroidota* ratio, aligned with the observations of Pan et al. in diabetic mice (Pan et al., 2024). Similar results were observed by Larsen et al. in patients with T2DM (Larsen et al., 2010). Specifically, OJO reduced the abundance of *Lactobacillus* and *Prevotella* while increasing *unclassified_f_*Lachnospiraceae, *Faecalibaculum*, UCG-005 (*P* < 0.05), Christensenellaceae*_R-7_group* (*P* < 0.05), *Romboutsia*, and *norank_f_norank_o_Clostridia_*UCG*-014* in T2DM rats. *Prevotella copri* is a dominant species within the genus *Prevotella*, which is more abundant in patients with insulin resistance and T2DM, leading to a decrease in insulin sensitivity through promoting BCAAs synthesis (Pedersen et al., 2016). *Prevotella* is a major genus responsible for acetate and propionate production (Fehlbaum et al., 2018; Hosmer et al., 2024). We also found that acetate and propionate were significantly increased in T2DM rats, which were notably reduced following OJO intervention. Although *Lactobacillus* is often regarded as a probiotic, its effects on patients with T2DM remain unclear due to inconsistent findings across studies (Gurung et al., 2020). However, some reports have observed a positive correlation between its abundance and T2DM, which aligns with our research findings (Wu et al., 2010; Forslund et al., 2015; Sedighi et al., 2017). The effect of *Lactobacillus* on T2DM might be strain- or species-specific, potentially offering an explanation for the inconsistent results at the genus level across different studies using this bacterium (Gurung et al., 2020). The abundant presence of *unclassified_f_*Lachnospiraceae in the gut benefits host health, as it degrades non-starch polysaccharides and produces butyrate, thereby improving glycaemic regulation (Ou et al., 2023). A large cross-sectional study involving 2,166 participants found that UCG-005 and Christensenellaceae*_R-7_group* were significantly associated with lower HOMA-IR, while *Romboutsia* was significantly associated with less T2DM, all these bacteria are butyrate producers, further supporting the notion that a rise in butyrate-producing bacteria correlates with a reduced risk of T2DM (Chen et al., 2021). Butyrate is thought to exert positive metabolic effects by enhancing mitochondrial activity, improving energy metabolism, activating intestinal gluconeogenesis, and preventing metabolic endotoxemia and inflammation through diverse mechanisms involving gene expression and hormonal regulation (Hartstra et al., 2015). However, our study did not observe an increase in butyrate with OJO treatment. This may be attributed to the fact that butyrate production is influenced by multiple factors, such as pH, substrate availability, redox conditions in the gut, and interactions between microbiota (Hamer et al., 2008; Louis and Flint, 2009). If the gut environment is unsuitable for the accumulation of butyrate, the actual production of butyrate may be inhibited despite an increase in the abundance of the relevant microbiota. Furthermore, in our study, the dominant genus in the MET group did not change significantly compared to the MOD group.

As the fermentation end products of functional oligosaccharides by gut microbiota, SCFAs influence glucose and lipid metabolism (Zhu et al., 2019). Acetate, propionate, and butyrate represent the primary SCFAs in the gut, accounting for nearly 95% of the total (Rios-Covian et al., 2016). Our results also showed that these three SCFAs were the most abundant. SCFAs are closely linked to T2DM, regulating multiple associated metabolic pathways, including modulating glucose metabolism, promoting fatty acid oxidation, and reducing inflammation, either by entering cells or by binding to cell-surface receptors like FFAR2, FFAR3, and GPR109A (Wu et al., 2023). Many studies have reported reduced acetate, propionate, and butyrate in diabetes (Wang et al., 2020; Yao et al., 2020; Fang et al., 2022). This investigation also showed a significant decrease in butyrate in T2DM rats, while acetate and propionate were notably increased. Ma et al. also reported a marked rise in acetate levels in T2DM rats, which may be attributed to insulin resistance and impaired glucose metabolism promoting cholesterol biosynthesis and lipogenesis, regulated through the conversion of acetate to acetyl-CoA (M. M. Ma and Mu, 2016). Additionally, the correlation between SCFAs and gut microbiota showed that acetate and propionate are positively linked to the *Prevotella* genus. The significant increase in *Prevotella* in T2DM rats may contribute to the elevated levels of acetate and propionate, whereas OJO intervention, which reduced the abundance of *Prevotella*, also markedly decreased acetate and propionate in T2DM rats. MET also significantly reduced acetate.

Metabolites derived from the gut microbiota, such as BAs, SCFAs, and BCAAs contribute to the regulation of host physiology (Wu et al., 2021; Wu et al., 2023). BAs are key signalling molecules that control glucose, fat, and energy metabolism by binding to the farnesoid X receptor and Takeda G protein-coupled receptor five in multiple organs, regulating incretin secretion, hepatic gluconeogenesis, glycogen synthesis, energy expenditure, inflammation, and gut microbiome composition, making them potential therapeutic targets for metabolic diseases (Shapiro et al., 2018; Xiang et al., 2021; Xie et al., 2021; Cai et al., 2022). Serum untargeted metabolomics identified 55 potential differential metabolites associated with diabetes, with bile acid metabolism emerging as an important mechanism through which OJO regulates glucose and lipid metabolism. We observed that both primary and secondary BAs were significantly elevated in T2DM rats. However, administering OJO led to a marked reduction in 12αOH BAs like cholic acid and deoxycholic acid, alongside an increase in the non-12αOH BA chenodeoxycholic acid. Recent studies have confirmed that the ratio of 12αOH BAs to non-12αOH BAs is a key determinant in metabolic diseases, including T2DM (Haeusler et al., 2013; Kaur et al., 2015; Zhong et al., 2022), obesity (Haeusler et al., 2016; Liu et al., 2024), non-alcoholic fatty liver disease (Hori et al., 2020; Lee et al., 2020), and hypercholesterolemia (Semova et al., 2022), with increased levels of 12αOH BAs correlate with T2DM, obesity, and insulin resistance. This is consistent with our findings. Significant enrichment in the primary bile acid biosynthesis pathway was also observed during the pathway analysis of differential metabolites. BAs are synthesized in the liver through the oxidation of cholesterol, mediated by cytochrome P450 enzymes. Cholesterol undergoes two biosynthetic pathways to generate the primary BAs, cholic acid and chenodeoxycholic acid (Jia et al., 2018; Jia et al., 2021). As a critical enzyme for cholic acid production, sterol-12α-hydroxylase (CYP8B1) regulates the 12α-hydroxylation of cholesterol metabolites, thereby determining the ratio of 12αOH BAs to non-12αOH BAs (Wahlstrom et al., 2016; Liu et al., 2024). We hypothesise that CYP8B1 inhibition in the liver of T2DM rats following OJO intervention leads to a reduction in cholic acid synthesis. The CYP8B1-cholic acid metabolic axis may represent a novel mechanism through which OJO improves glucose and lipid metabolism disorders, warranting further research to confirm this. Correlation analysis also revealed a negative association between these BAs and *unclassified_f_*Lachnospiraceae. We also observed that OJO lowered the increased levels of L-phenylalanine and L-tyrosine in diabetic rats while restoring the reduced levels of cinnamic acid and 2-hydroxycinnamic acid. Enrichment analysis further indicated that the primary metabolic pathways involved were phenylalanine, tyrosine, and tryptophan biosynthesis, as well as phenylalanine metabolism. Aromatic amino acids phenylalanine and tyrosine, similar to BCAAs, have been shown to have a positive correlation with the risk of T2DM (Wang et al., 2011). Elevated plasma levels of phenylalanine and tyrosine were observed in prediabetic, and T2DM patients, and these amino acid levels correlate with the degree of insulin resistance (Guasch-Ferre et al., 2016). Cinnamic acid and its derivatives have been shown to contribute to the prevention and management of diabetes and its complications, through mechanisms such as stimulating insulin secretion, improving pancreatic β-cell function, inhibiting hepatic gluconeogenesis, enhancing glucose uptake, and increasing insulin signalling pathways (Adisakwattana, 2017). These findings suggest that OJO exerts anti-T2DM effects by improving dysregulated glucose and lipid metabolism by regulating BAs and aromatic amino acids synthesis and metabolism. However, additional studies are required to clarify the mechanisms involved. Overall, this study has several limitations. First, the long-term persistence of the beneficial effects of OJO after discontinuation of treatment was not assessed, and thus its sustained efficacy remains uncertain. Second, although our results demonstrate associations between OJO-induced modulation of gut microbiota, serum metabolites, and improved glucose metabolism, causal relationships were not experimentally validated; in particular, fecal microbiota transplantation or targeted metabolite intervention experiments were not performed.

## 5 Conclusion

In summary, this study demonstrates that OJO exhibits significant protective effects in rats with T2DM induced by HFD/STZ. OJO treatment significantly improved glucose and lipid metabolism disorders, alleviated insulin resistance, and mitigated diabetic symptoms such as polyuria and weight loss in T2DM rats. The anti-T2DM mechanism of OJO may be associated with improving gut microbiota dysbiosis and regulating the levels of BAs, aromatic amino acids, and SCFAs. These findings suggest that OJO has prebiotic properties and holds great promise as a functional food with anti-T2DM potential. We plan to conduct further studies on how SCFAs, BAs, and aromatic amino acids contribute to the adjustment of glucose and lipid metabolism by OJO, providing experimental support for its use in preventing and treating T2DM.

## Data Availability

The original contributions presented in the study are publicly available. The 16S rRNA sequencing data can be found in the NCBI Sequence Read Archive under accession number PRJNA1359156.

## References

[B1] Adisakwattana S. (2017). Cinnamic acid and its derivatives: mechanisms for prevention and management of diabetes and its complications. Nutrients 9 (2), 163. 10.3390/nu9020163 28230764 PMC5331594

[B2] Araiza-Saldana C. I. Pedraza-Priego E. F. Torres-Lopez J. E. Rocha-Gonzalez H. I. Castaneda-Corral G. Hong-Chong E. (2015). Fosinopril prevents the development of tactile allodynia in a streptozotocin-induced diabetic Rat model. Drug Dev. Res. 76 (8), 442–449. 10.1002/ddr.21280 26349482

[B3] Baars D. P. Fondevila M. F. Meijnikman A. S. Nieuwdorp M. (2024). The central role of the gut microbiota in the pathophysiology and management of type 2 diabetes. Cell Host Microbe 32 (8), 1280–1300. 10.1016/j.chom.2024.07.017 39146799

[B4] Bai R. Cui F. Li W. Wang Y. Wang Z. Gao Y. (2022). Codonopsis pilosula oligosaccharides modulate the gut microbiota and change serum metabolomic profiles in high-fat diet-induced obese mice. Food Funct. 13 (15), 8143–8157. 10.1039/d2fo01119k 35816111

[B5] Cai J. Rimal B. Jiang C. Chiang J. Y. L. Patterson A. D. (2022). Bile acid metabolism and signaling, the microbiota, and metabolic disease. Pharmacol. Ther. 237, 108238. 10.1016/j.pharmthera.2022.108238 35792223

[B6] Cao X. Wang X. Ren Y. Sun Y. Yang Z. Ge J. (2023). Lonicera caerulea L. polyphenols improve short-chain fatty acid levels by reshaping the microbial structure of fermented feces *in vitro* . Front. Microbiol. 14, 1228700. 10.3389/fmicb.2023.1228700 37965545 PMC10641692

[B7] Chao P. C. Li Y. Chang C. H. Shieh J. P. Cheng J. T. Cheng K. C. (2018). Investigation of insulin resistance in the popularly used four rat models of type-2 diabetes. Biomed. Pharmacother. 101, 155–161. 10.1016/j.biopha.2018.02.084 29486333

[B8] Chen X. Tang J. Xie W. Wang J. Jin J. Ren J. (2013). Protective effect of the polysaccharide from Ophiopogon japonicus on streptozotocin-induced diabetic rats. Carbohydr. Polym. 94 (1), 378–385. 10.1016/j.carbpol.2013.01.037 23544552

[B9] Chen M. H. Chen X. J. Wang M. Lin L. G. Wang Y. T. (2016). Ophiopogon japonicus--A phytochemical, ethnomedicinal and pharmacological review. J. Ethnopharmacol. 181, 193–213. 10.1016/j.jep.2016.01.037 26826325

[B10] Chen Z. Radjabzadeh D. Chen L. Kurilshikov A. Kavousi M. Ahmadizar F. (2021). Association of insulin resistance and type 2 diabetes with gut microbial diversity: a microbiome-wide analysis from population studies. JAMA Netw. Open 4 (7), e2118811. 10.1001/jamanetworkopen.2021.18811 34323983 PMC8322996

[B11] Chen B. Li Z. Fang J. Liu Y. Lin A. (2024). Oligosaccharides of Ophiopogon japonicus ameliorate insulin resistance and glucolipid metabolism in HFD/STZ-induced T2DM rats and IR-HepG2 cells *via* activation of the IRS-1/PI3K/AKT/GSK-3β pathway. J. Funct. Foods 120, 106368. 10.1016/j.jff.2024.106368

[B12] China N. H. C (2024). Announcement no. 4 of 2024: addition of four substances to the list of substances traditionally used as both food and Chinese medicine. Available online at: https://www.nhc.gov.cn/sps/c100088/202408/53150d0918ec40899b5293147ec0dd01.shtml (Accessed October 19, 2025).

[B13] Ding L. Li P. Lau C. B. Chan Y. W. Xu D. Fung K. P. (2012). Mechanistic studies on the antidiabetic activity of a polysaccharide-rich extract of Radix Ophiopogonis. Phytother. Res. 26 (1), 101–105. 10.1002/ptr.3505 21560174

[B14] Du L. Li Q. Yi H. Kuang T. Tang Y. Fan G. (2022). Gut microbiota-derived metabolites as key actors in type 2 diabetes mellitus. Biomed. Pharmacother. 149, 112839. 10.1016/j.biopha.2022.112839 35325852

[B15] Fang J. Wang X. Lu M. He X. Yang X. (2018). Recent advances in polysaccharides from Ophiopogon japonicus and Liriope spicata var. prolifera. Int. J. Biol. Macromol. 114, 1257–1266. 10.1016/j.ijbiomac.2018.04.022 29634971

[B16] Fang J. Lin Y. Xie H. Farag M. A. Feng S. Li J. (2022). Dendrobium officinale leaf polysaccharides ameliorated hyperglycemia and promoted gut bacterial associated SCFAs to alleviate type 2 diabetes in adult mice. Food Chem. X 13, 100207. 10.1016/j.fochx.2022.100207 35498995 PMC9039915

[B17] Fehlbaum S. Prudence K. Kieboom J. Heerikhuisen M. van den Broek T. Schuren F. H. J. (2018). *In vitro* fermentation of selected prebiotics and their effects on the composition and activity of the adult Gut Microbiota. Int. J. Mol. Sci. 19 (10), 3097. 10.3390/ijms19103097 30308944 PMC6213619

[B18] Forslund K. Hildebrand F. Nielsen T. Falony G. Le Chatelier E. Sunagawa S. (2015). Disentangling type 2 diabetes and metformin treatment signatures in the human gut microbiota. Nature 528 (7581), 262–266. 10.1038/nature15766 26633628 PMC4681099

[B19] Garcia-Gutierrez E. O'Mahony A. K. Dos Santos R. S. Marroqui L. Cotter P. D. (2024). Gut microbial metabolic signatures in diabetes mellitus and potential preventive and therapeutic applications. Gut Microbes 16 (1), 2401654. 10.1080/19490976.2024.2401654 39420751 PMC11492678

[B20] Guasch-Ferre M. Hruby A. Toledo E. Clish C. B. Martinez-Gonzalez M. A. Salas-Salvado J. (2016). Metabolomics in prediabetes and diabetes: a systematic review and meta-analysis. Diabetes Care 39 (5), 833–846. 10.2337/dc15-2251 27208380 PMC4839172

[B21] Gurung M. Li Z. You H. Rodrigues R. Jump D. B. Morgun A. (2020). Role of gut microbiota in type 2 diabetes pathophysiology. EBioMedicine 51, 102590. 10.1016/j.ebiom.2019.11.051 31901868 PMC6948163

[B22] Haeusler R. A. Astiarraga B. Camastra S. Accili D. Ferrannini E. (2013). Human insulin resistance is associated with increased plasma levels of 12α-hydroxylated bile acids. Diabetes 62 (12), 4184–4191. 10.2337/db13-0639 23884887 PMC3837033

[B23] Haeusler R. A. Camastra S. Nannipieri M. Astiarraga B. Castro-Perez J. Xie D. (2016). Increased bile acid synthesis and impaired bile acid transport in human obesity. J. Clin. Endocrinol. Metab. 101 (5), 1935–1944. 10.1210/jc.2015-2583 26684275 PMC4870845

[B24] Hamer H. M. Jonkers D. Venema K. Vanhoutvin S. Troost F. J. Brummer R. J. (2008). Review article: the role of butyrate on colonic function. Aliment. Pharmacol. Ther. 27 (2), 104–119. 10.1111/j.1365-2036.2007.03562.x 17973645

[B25] Hartstra A. V. Bouter K. E. Backhed F. Nieuwdorp M. (2015). Insights into the role of the microbiome in obesity and type 2 diabetes. Diabetes Care 38 (1), 159–165. 10.2337/dc14-0769 25538312

[B26] Hori S. Abe T. Lee D. G. Fukiya S. Yokota A. Aso N. (2020). Association between 12α-hydroxylated bile acids and hepatic steatosis in rats fed a high-fat diet. J. Nutr. Biochem. 83, 108412. 10.1016/j.jnutbio.2020.108412 32534424

[B27] Hosmer J. McEwan A. G. Kappler U. (2024). Bacterial acetate metabolism and its influence on human epithelia. Emerg. Top. Life Sci. 8 (1), 1–13. 10.1042/ETLS20220092 36945843 PMC10903459

[B28] Illiano P. Brambilla R. Parolini C. (2020). The mutual interplay of gut microbiota, diet and human disease. FEBS J. 287 (5), 833–855. 10.1111/febs.15217 31955527

[B29] Jia W. Xie G. Jia W. (2018). Bile acid-microbiota crosstalk in gastrointestinal inflammation and carcinogenesis. Nat. Rev. Gastroenterol. Hepatol. 15 (2), 111–128. 10.1038/nrgastro.2017.119 29018272 PMC5899973

[B30] Jia W. Wei M. Rajani C. Zheng X. (2021). Targeting the alternative bile acid synthetic pathway for metabolic diseases. Protein Cell 12 (5), 411–425. 10.1007/s13238-020-00804-9 33252713 PMC8106556

[B31] Kaur A. Patankar J. V. de Haan W. Ruddle P. Wijesekara N. Groen A. K. (2015). Loss of Cyp8b1 improves glucose homeostasis by increasing GLP-1. Diabetes 64 (4), 1168–1179. 10.2337/db14-0716 25338812

[B32] Kim J. Noh W. Kim A. Choi Y. Kim Y. S. (2023). The effect of fenugreek in type 2 diabetes and prediabetes: a systematic review and meta-analysis of randomized controlled trials. Int. J. Mol. Sci. 24 (18), 13999. 10.3390/ijms241813999 37762302 PMC10531284

[B33] Kitahiro Y. Koike A. Sonoki A. Muto M. Ozaki K. Shibano M. (2018). Anti-inflammatory activities of Ophiopogonis Radix on hydrogen peroxide-induced cellular senescence of normal human dermal fibroblasts. J. Nat. Med. 72 (4), 905–914. 10.1007/s11418-018-1223-9 29961188

[B34] Kumar S. Mittal A. Babu D. Mittal A. (2021). Herbal medicines for diabetes management and its secondary complications. Curr. Diabetes Rev. 17 (4), 437–456. 10.2174/1573399816666201103143225 33143632

[B35] Larsen N. Vogensen F. K. van den Berg F. W. Nielsen D. S. Andreasen A. S. Pedersen B. K. (2010). Gut microbiota in human adults with type 2 diabetes differs from non-diabetic adults. PLoS One 5 (2), e9085. 10.1371/journal.pone.0009085 20140211 PMC2816710

[B36] Le Chatelier E. Nielsen T. Qin J. Prifti E. Hildebrand F. Falony G. (2013). Richness of human gut microbiome correlates with metabolic markers. Nature 500 (7464), 541–546. 10.1038/nature12506 23985870

[B37] Lee J. Y. Shimizu H. Hagio M. Fukiya S. Watanabe M. Tanaka Y. (2020). 12α-Hydroxylated bile acid induces hepatic steatosis with dysbiosis in rats. Biochim. Biophys. Acta Mol. Cell Biol. Lipids 1865 (12), 158811. 10.1016/j.bbalip.2020.158811 32896622

[B38] Li S. Z. (1596). Compendium of Materia Medica. Beijing: People’s Health Publishing House.

[B39] Li P. B. Lin W. L. Wang Y. G. Peng W. Cai X. Y. Su W. W. (2012). Antidiabetic activities of oligosaccharides of Ophiopogonis japonicus in experimental type 2 diabetic rats. Int. J. Biol. Macromol. 51 (5), 749–755. 10.1016/j.ijbiomac.2012.07.007 22800731

[B40] Li C. N. Wang X. Lei L. Liu M. Z. Li R. C. Sun S. J. (2020). Berberine combined with stachyose induces better glycometabolism than berberine alone through modulating gut microbiota and fecal metabolomics in diabetic mice. Phytother. Res. 34 (5), 1166–1174. 10.1002/ptr.6588 31833107 PMC7216932

[B41] Lin W. L. Su W. W. Cai X. Y. Luo L. K. Li P. B. Wang Y. G. (2011). Fermentation effects of oligosaccharides of Radix Ophiopogonis on alloxan-induced diabetes in mice. Int. J. Biol. Macromol. 49 (2), 194–200. 10.1016/j.ijbiomac.2011.04.011 21549746

[B42] Liu H. Zhang Z. Li J. Liu W. Warda M. Cui B. (2022). Oligosaccharides derived from Lycium barbarum ameliorate glycolipid metabolism and modulate the gut microbiota community and the faecal metabolites in a type 2 diabetes mouse model: metabolomic bioinformatic analysis. Food Funct. 13 (9), 5416–5429. 10.1039/d1fo02667d 35475434

[B43] Liu Y. Tu J. Shi L. Fang Z. Fan M. Zhang J. (2024). CYP8B1 downregulation mediates the metabolic effects of vertical sleeve gastrectomy in mice. Hepatology 79 (5), 1005–1018. 10.1097/HEP.0000000000000627 37820064 PMC11006827

[B44] Louis P. Flint H. J. (2009). Diversity, metabolism and microbial ecology of butyrate-producing bacteria from the human large intestine. FEMS Microbiol. Lett. 294 (1), 1–8. 10.1111/j.1574-6968.2009.01514.x 19222573

[B45] Lu X. Tong W. Wang S. Li J. Zheng J. Fan X. (2017). Comparison of the chemical consituents and immunomodulatory activity of ophiopogonis radix from two different producing areas. J. Pharm. Biomed. Anal. 134, 60–70. 10.1016/j.jpba.2016.11.025 27875789

[B46] Ma M. M. Mu T. H. (2016). Anti-diabetic effects of soluble and insoluble dietary fibre from deoiled cumin in low-dose streptozotocin and high glucose-fat diet-induced type 2 diabetic rats. J. Funct. Foods 25, 186–196. 10.1016/j.jff.2016.05.011

[B47] Ma Q. Li Y. Li P. Wang M. Wang J. Tang Z. (2019). Research progress in the relationship between type 2 diabetes mellitus and intestinal flora. Biomed. Pharmacother. 117, 109138. 10.1016/j.biopha.2019.109138 31247468

[B48] Majety P. Lozada Orquera F. A. Edem D. Hamdy O. (2023). Pharmacological approaches to the prevention of type 2 diabetes mellitus. Front. Endocrinol. (Lausanne) 14, 1118848. 10.3389/fendo.2023.1118848 36967777 PMC10033948

[B49] Miaffo D. Ntchapda F. Mahamad T. A. Maidadi B. Kamanyi A. (2021). Hypoglycemic, antidyslipidemic and antioxydant effects of Vitellaria paradoxa barks extract on high-fat diet and streptozotocin-induced type 2 diabetes rats. Metabol. Open 9, 100071. 10.1016/j.metop.2020.100071 33364595 PMC7750157

[B50] Ou Y. Guo Y. Chen M. Lu X. Guo Z. Zheng B. (2023). Gut microbiome-serum metabolic profiles: insight into the hypoglycemic effect of Porphyra haitanensis glycoprotein on hyperglycemic mice. Food Funct. 14 (17), 7977–7991. 10.1039/d3fo02040a 37578326

[B51] Pan Z. Luo H. He F. Du Y. Wang J. Zeng H. (2024). Guava polysaccharides attenuate high fat and STZ-induced hyperglycemia by regulating gut microbiota and arachidonic acid metabolism. Int. J. Biol. Macromol. 276 (Pt 1), 133725. 10.1016/j.ijbiomac.2024.133725 38986994

[B52] Pedersen H. K. Gudmundsdottir V. Nielsen H. B. Hyotylainen T. Nielsen T. Jensen B. A. (2016). Human gut microbes impact host serum metabolome and insulin sensitivity. Nature 535 (7612), 376–381. 10.1038/nature18646 27409811

[B53] Qin J. Li Y. Cai Z. Li S. Zhu J. Zhang F. (2012). A metagenome-wide association study of gut microbiota in type 2 diabetes. Nature 490 (7418), 55–60. 10.1038/nature11450 23023125

[B54] Ren Y. Bai Y. Zhang Z. Cai W. Del Rio Flores A. (2019). The preparation and structure analysis methods of natural polysaccharides of plants and fungi: a review of recent development. Molecules 24 (17), 3122. 10.3390/molecules24173122 31466265 PMC6749352

[B55] Rios-Covian D. Ruas-Madiedo P. Margolles A. Gueimonde M. de Los Reyes-Gavilan C. G. Salazar N. (2016). Intestinal short chain fatty acids and their link with diet and human health. Front. Microbiol. 7, 185. 10.3389/fmicb.2016.00185 26925050 PMC4756104

[B56] Roy N. K. Parama D. Banik K. Bordoloi D. Devi A. K. Thakur K. K. (2019). An update on pharmacological potential of boswellic acids against chronic diseases. Int. J. Mol. Sci. 20 (17), 4101. 10.3390/ijms20174101 31443458 PMC6747466

[B57] Sato J. Kanazawa A. Ikeda F. Yoshihara T. Goto H. Abe H. (2014). Gut dysbiosis and detection of “live gut bacteria” in blood of Japanese patients with type 2 diabetes. Diabetes Care 37 (8), 2343–2350. 10.2337/dc13-2817 24824547

[B58] Sedighi M. Razavi S. Navab-Moghadam F. Khamseh M. E. Alaei-Shahmiri F. Mehrtash A. (2017). Comparison of gut microbiota in adult patients with type 2 diabetes and healthy individuals. Microb. Pathog. 111, 362–369. 10.1016/j.micpath.2017.08.038 28912092

[B59] Semova I. Levenson A. E. Krawczyk J. Bullock K. Gearing M. E. Ling A. V. (2022). Insulin prevents hypercholesterolemia by suppressing 12α-Hydroxylated bile acids. Circulation 145 (13), 969–982. 10.1161/CIRCULATIONAHA.120.045373 35193378 PMC9365453

[B60] Shapiro H. Kolodziejczyk A. A. Halstuch D. Elinav E. (2018). Bile acids in glucose metabolism in health and disease. J. Exp. Med. 215 (2), 383–396. 10.1084/jem.20171965 29339445 PMC5789421

[B61] Sircana A. Framarin L. Leone N. Berrutti M. Castellino F. Parente R. (2018). Altered gut microbiota in type 2 diabetes: just a coincidence? Curr. Diab Rep. 18 (10), 98. 10.1007/s11892-018-1057-6 30215149

[B62] Song Y. Wu M. S. Tao G. Lu M. W. Lin J. Huang J. Q. (2020). Feruloylated oligosaccharides and ferulic acid alter gut microbiome to alleviate diabetic syndrome. Food Res. Int. 137, 109410. 10.1016/j.foodres.2020.109410 33233097

[B63] Srinivasan K. Viswanad B. Asrat L. Kaul C. L. Ramarao P. (2005). Combination of high-fat diet-fed and low-dose streptozotocin-treated rat: a model for type 2 diabetes and pharmacological screening. Pharmacol. Res. 52 (4), 313–320. 10.1016/j.phrs.2005.05.004 15979893

[B64] Sun H. Saeedi P. Karuranga S. Pinkepank M. Ogurtsova K. Duncan B. B. (2022). IDF Diabetes Atlas: global, regional and country-level diabetes prevalence estimates for 2021 and projections for 2045. Diabetes Res. Clin. Pract. 183, 109119. 10.1016/j.diabres.2021.109119 34879977 PMC11057359

[B65] The Lancet (2017). Diabetes: a dynamic disease. Lancet 389 (10085), 2163. 10.1016/S0140-6736(17)31537-4 28589879

[B66] Wahlstrom A. Sayin S. I. Marschall H. U. Backhed F. (2016). Intestinal crosstalk between bile acids and microbiota and its impact on host metabolism. Cell Metab. 24 (1), 41–50. 10.1016/j.cmet.2016.05.005 27320064

[B67] Wang T. J. Larson M. G. Vasan R. S. Cheng S. Rhee E. P. McCabe E. (2011). Metabolite profiles and the risk of developing diabetes. Nat. Med. 17 (4), 448–453. 10.1038/nm.2307 21423183 PMC3126616

[B68] Wang L. Y. Wang Y. Xu D. S. Ruan K. F. Feng Y. Wang S. (2012). MDG-1, a polysaccharide from Ophiopogon japonicus exerts hypoglycemic effects through the PI3K/Akt pathway in a diabetic KKAy mouse model. J. Ethnopharmacol. 143 (1), 347–354. 10.1016/j.jep.2012.06.050 22776833

[B69] Wang Y. Dilidaxi D. Wu Y. Sailike J. Sun X. Nabi X. H. (2020). Composite probiotics alleviate type 2 diabetes by regulating intestinal microbiota and inducing GLP-1 secretion in db/db mice. Biomed. Pharmacother. 125, 109914. 10.1016/j.biopha.2020.109914 32035395

[B70] Wu X. Ma C. Han L. Nawaz M. Gao F. Zhang X. (2010). Molecular characterisation of the faecal microbiota in patients with type II diabetes. Curr. Microbiol. 61 (1), 69–78. 10.1007/s00284-010-9582-9 20087741

[B71] Wu W. Z. Su W. W. Wang Y. G. Peng W. Wu Z. Li P. B. (2017). The hypoglycaemic effect of Ophiopogonis japonicus oligosaccharide on db/db diabetic mice. Acta Sci. Nat. Univ. Sunyatseni 56 (6), 128–133. 10.13471/j.cnki.acta.snus.2017.06.020

[B72] Wu J. Wang K. Wang X. Pang Y. Jiang C. (2021). The role of the gut microbiome and its metabolites in metabolic diseases. Protein Cell 12 (5), 360–373. 10.1007/s13238-020-00814-7 33346905 PMC8106557

[B73] Wu J. Yang K. Fan H. Wei M. Xiong Q. (2023). Targeting the gut microbiota and its metabolites for type 2 diabetes mellitus. Front. Endocrinol. (Lausanne) 14, 1114424. 10.3389/fendo.2023.1114424 37229456 PMC10204722

[B74] Xiang J. Zhang Z. Xie H. Zhang C. Bai Y. Cao H. (2021). Effect of different bile acids on the intestine through enterohepatic circulation based on FXR. Gut Microbes 13 (1), 1949095. 10.1080/19490976.2021.1949095 34313539 PMC8346203

[B75] Xie A. J. Mai C. T. Zhu Y. Z. Liu X. C. Xie Y. (2021). Bile acids as regulatory molecules and potential targets in metabolic diseases. Life Sci. 287, 120152. 10.1016/j.lfs.2021.120152 34793769

[B76] Xu D. Z. Su W. W. Wang Y. G. Peng W. Wu Z. Li P. B. (2017). Extraction、purification and α-glucosidase inhibitory activity of oligosaccharide from *Ophiopogonis japonicas* . China Med. Her. 14, 20–22. 10.13604/j.cnki.46-1064/r.2017.02.05

[B77] Xu L. Li Y. Dai Y. Peng J. (2018). Natural products for the treatment of type 2 diabetes mellitus: pharmacology and mechanisms. Pharmacol. Res. 130, 451–465. 10.1016/j.phrs.2018.01.015 29395440

[B78] Yao Y. Yan L. Chen H. Wu N. Wang W. Wang D. (2020). Cyclocarya paliurus polysaccharides alleviate type 2 diabetic symptoms by modulating gut microbiota and short-chain fatty acids. Phytomedicine 77, 153268. 10.1016/j.phymed.2020.153268 32663709

[B79] Zhang Y. Chen B. Zhang H. Zhang J. Xue J. (2024). Extraction, purification, structural characterization, bioactivities, modifications and structure-activity relationship of polysaccharides from Ophiopogon japonicus: a review. Front. Nutr. 11, 1484865. 10.3389/fnut.2024.1484865 39624687 PMC11610408

[B80] Zheng Y. Ley S. H. Hu F. B. (2018). Global aetiology and epidemiology of type 2 diabetes mellitus and its complications. Nat. Rev. Endocrinol. 14 (2), 88–98. 10.1038/nrendo.2017.151 29219149

[B81] Zheng Y. Bai L. Zhou Y. Tong R. Zeng M. Li X. (2019). Polysaccharides from Chinese herbal medicine for anti-diabetes recent advances. Int. J. Biol. Macromol. 121, 1240–1253. 10.1016/j.ijbiomac.2018.10.072 30342938

[B82] Zhong S. Chevre R. Castano Mayan D. Corliano M. Cochran B. J. Sem K. P. (2022). Haploinsufficiency of CYP8B1 associates with increased insulin sensitivity in humans. J. Clin. Invest 132 (21), e152961. 10.1172/JCI152961 36107630 PMC9621133

[B83] Zhu D. Yan Q. Liu J. Wu X. Jiang Z. (2019). Can functional oligosaccharides reduce the risk of diabetes mellitus? FASEB J. 33 (11), 11655–11667. 10.1096/fj.201802802RRR 31415188 PMC6902710

[B84] Zhu L. Sha L. Li K. Wang Z. Wang T. Li Y. (2020). Dietary flaxseed oil rich in omega-3 suppresses severity of type 2 diabetes mellitus *via* anti-inflammation and modulating gut microbiota in rats. Lipids Health Dis. 19 (1), 20. 10.1186/s12944-019-1167-4 32028957 PMC7006389

[B85] Zuo W. F. Pang Q. Yao L. P. Zhang Y. Peng C. Huang W. (2023). Gut microbiota: a magical multifunctional target regulated by medicine food homology species. J. Adv. Res. 52, 151–170. 10.1016/j.jare.2023.05.011 37269937 PMC10555941

