# Supplementary material for: Effects of Ophiopogon japonicus oligosaccharides on type 2 diabetes in rats via modulation of gut microbiota and metabolites
Source: Front Pharmacol. 2025 Nov 20;16:1710883. doi: 10.3389/fphar.2025.1710883 (PMC12675414; doi:10.3389/fphar.2025.1710883)
Supplement: Supplementary file 1 [file DataSheet1.docx]

Supplementary Material

# Supplementary Materials and methods

- 1. **Animals**

The high-fat diet was composed of 20% sugar, 15% lard, 1.2% cholesterol, 0.2% sodium cholate, 10% casein, 0.6% calcium hydrogen phosphate, 0.4% stone powder, 0.4% premix, and 52.2% basal diet. It was provided by Guangdong Medical Laboratory Animal Center (licence number: Guangdong Feeding Certificate (2019) 05073).

## Serum untargeted metabolomics analysis

Serum sample analysis using UHPLC-QTOF-MS was performed with the Ultimate 3000 UHPLC system and Q-Exactive Focus Quadrupole-Orbitrap Mass Spectrometer (Thermo Fisher Scientific, USA). A Waters CORTECS UPLC C18+ column (2.1 × 100 mm, 1.6 μm) facilitated the separation with a gradient elution of 0.1% formic acid in water (A) and methanol (B): 0–5 min, 5% B; 5–9 min, 5%–30% B; 9–15 min, 30%–60% B; 15–25 min, 60%–95% B; 25–26 min, 95% B; 26–27 min, 95%–5% B; 27–30 min, 5% B. The flow rate was fixed at 0.2 mL·min-1, with the column maintained at 35 °C. Detection was achieved employing an electrospray ionisation source operating in negative and positive modes. The ion transfer tube was heated to 320 °C, with capillary temperatures set to 350 °C (+) and 300 °C (−), and spray voltage configured to 3.5 kV (+) and 3.0 kV (−). The Full MS/dd-MS2 scanning method was employed, with the MS1 full scan spanning m/z 70 to 1050 and a resolution of 70,000. The MS2 scan was conducted at a resolution of 17500, using higher-energy collisional dissociation as the fragmentation method, with normalised collision energies set to 20, 40, and 60.

# Supplementary Figures and Tables


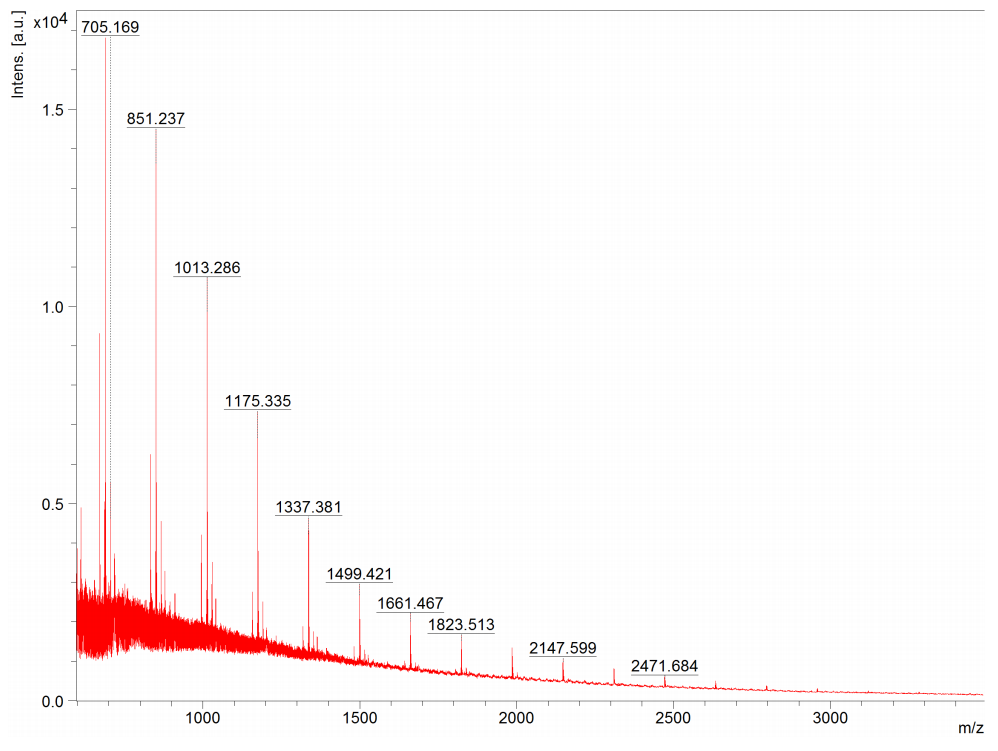


**Figure S1 MALDI-TOF mass spectrum of OJO**.


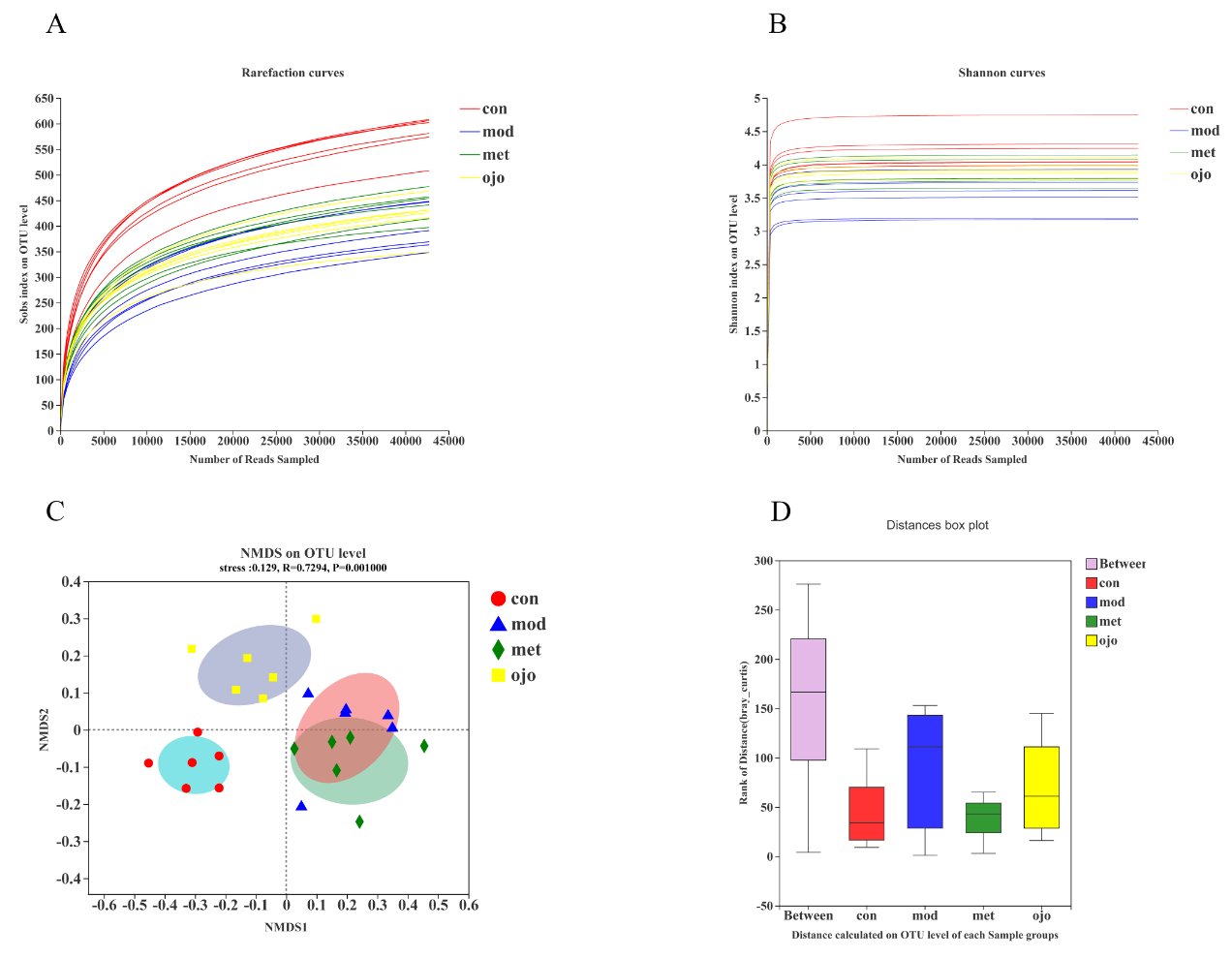


**Figure S2 Analysis of gut microbiota α-diversity and β-diversity. (A)** Rarefaction curves; **(B)** Shannon curves; **(C)** NMDS analysis; **(D)** ANOSIM analysis. ^*^*P* < 0.05, ^**^*P* < 0.01 vs MOD.


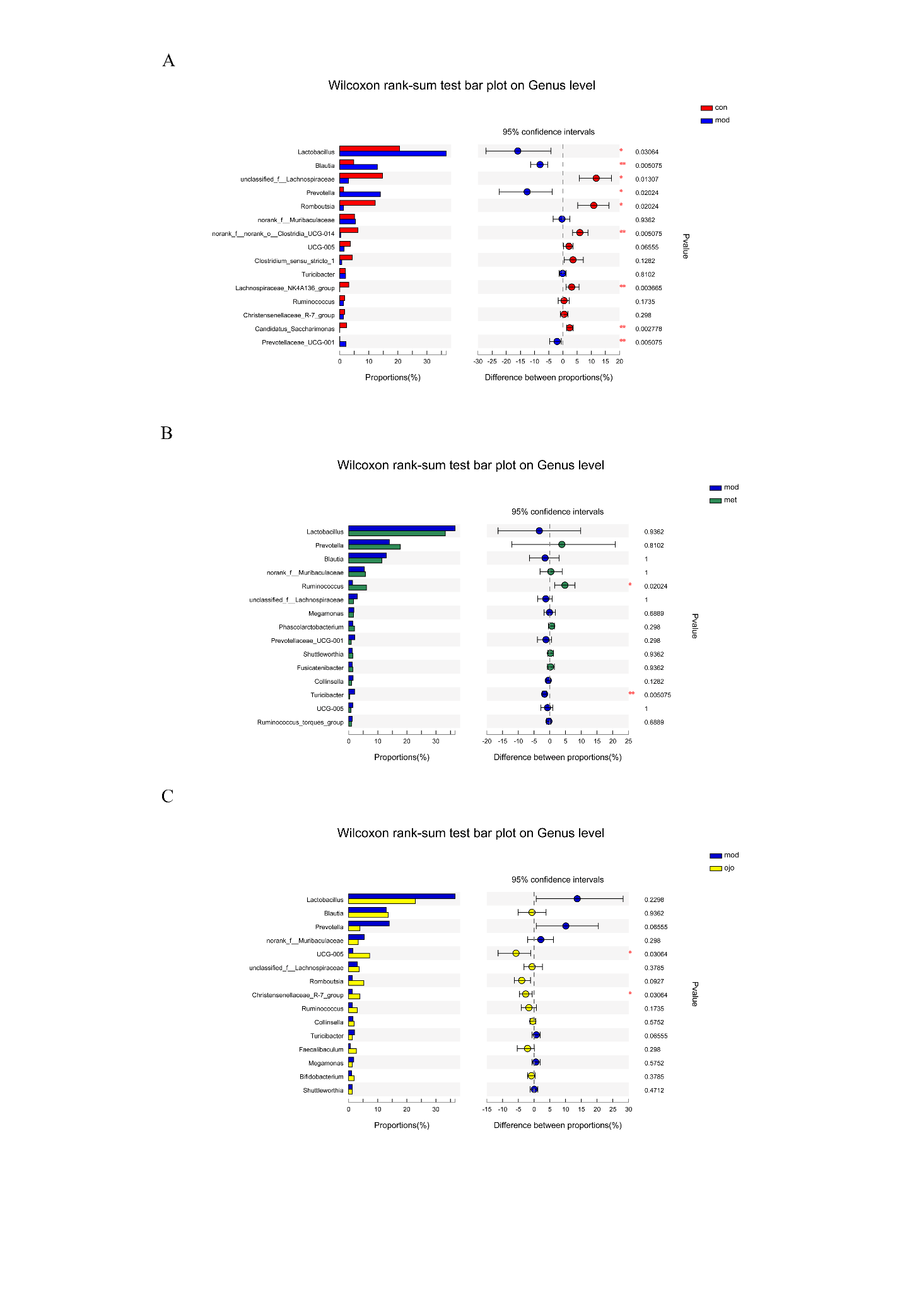


**Figure S3 Differential analysis of gut microbiota at the genus level. (A)** comparison between the CON group and the MOD group; **(B)** comparison between the MOD group and the MET group; **(C)** comparison between the MOD group and the H-OJO group. ^*^*P* < 0.05, ^**^*P* < 0.01, ^***^*P* < 0.001.


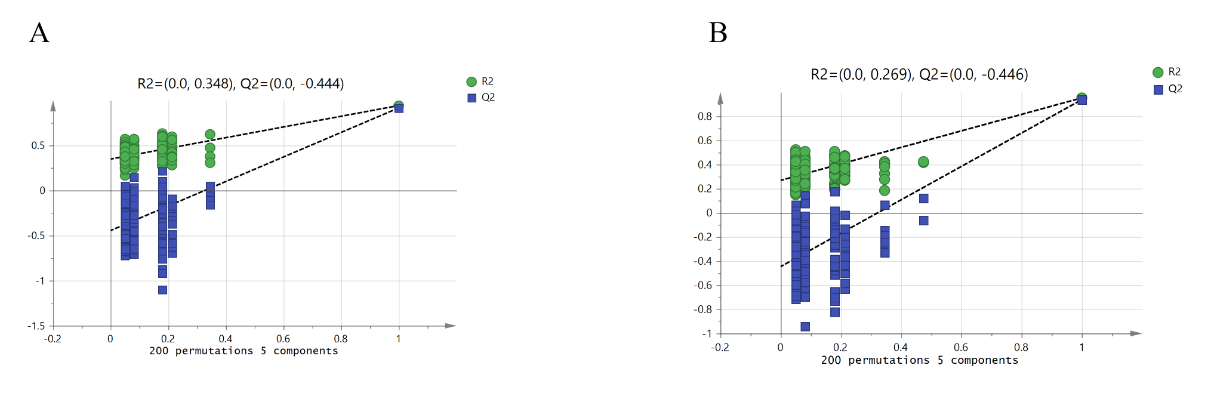


**Figure S4 Permutation test analysis of the OPLS-DA model (n = 200) under (A) positive ion mode and (B) negative ion mode**.

**Table S1 OJO altered differential metabolites of serum in T2DM rats**

| Metabolites | tR/min | m/z | formula | mode | HMDB ID | Trend | | | | |
| --- | --- | --- | --- | --- | --- | --- | --- | --- | --- | --- |
|  |  |  |  |  |  | MOD/CON | H/  MOD | L/  MOD | M/  MOD | MET/  MOD |
| L-Tryptophan | 8.98 | 204.0898 | C11H12N2O2 | + | HMDB0013609 | ↑ |  |  |  |  |
| Indoleacrylic acid | 8.98 | 187.0633 | C11H9NO2 | + | HMDB0000734 | ↓ |  |  |  |  |
| Cinnamic acid | 4.28 | 148.0524 | C9H8O2 | + | HMDB0000567 | ↓ | ↑ | ↑ | ↑ |  |
| L-Phenylalanine | 4.28 | 165.0789 | C9H11NO2 | + | HMDB0000159 | ↑ | ↓ | ↓ | ↓ |  |
| Glycocholic acid | 22.25 | 465.3084 | C26H43NO6 | + | HMDB0000138 | ↑ | ↓ | ↓ |  |  |
| 12-Ketodeoxycholic acid | 23.77 | 390.2763 | C24H38O4 | + | HMDB0000328 | ↑ | ↓ | ↓ |  |  |
| 2-Hydroxycinnamic acid | 2.40 | 164.0475 | C9H8O3 | + | HMDB0002641 | ↓ | ↑ | ↑ |  |  |
| LysoPC (16:0) | 26.88 | 495.3314 | C24H50NO7P | + | HMDB0010382 | ↓ | ↑ |  | ↑ | ↑ |
| Cervonoyl ethanolamide | 21.76 | 372.2659 | C24H36O3 | + | HMDB0013627 | ↑ |  | ↓ |  |  |
| LysoPC (16:1) | 26.15 | 493.3157 | C24H48NO7P | + | HMDB0010383 | ↓ | ↑ | ↑ | ↑ | ↑ |
| LysoPE (P-18:1/0:0) | 19.85 | 463.2930 | C23H46NO6P | + | HMDB0240599 | ↑ |  |  | ↓ |  |
| Succinylacetoacetate | 2.10 | 202.0454 | C8H10O6 | + | HMDB0240258 | ↑ | ↓ | ↓ |  |  |
| 3b-Hydroxy-5-cholenoic acid | 25.30 | 374.2814 | C24H38O3 | + | HMDB0000308 | ↑ |  | ↓ |  |  |
| Tetracosahexaenoic acid | 25.30 | 356.2710 | C24H36O2 | + | HMDB0002007 | ↑ |  | ↓ |  |  |
| 7-ketodeoxycholic acid | 21.88 | 406.2717 | C24H38O5 | + | HMDB0000391 | ↑ |  | ↓ |  |  |
| N-Lactoylleucine | 1.97 | 203.1154 | C9H17NO4 | + | HMDB0062176 | ↑ | ↓ |  |  | ↓ |
| L-Tyrosine | 2.16 | 181.0739 | C9H11NO3 | + | HMDB0000158 | ↑ | ↓ | ↓ |  |  |
| LysoPC (20:4) | 26.37 | 543.3309 | C28H50NO7P | + | HMDB0010396 | ↓ |  |  | ↑ | ↑ |
| LysoPC (18:3) | 26.89 | 517.3135 | C26H48NO7P | + | HMDB0010387 | ↓ |  |  | ↑ | ↑ |
| Taurocholic acid | 26.15 | 515.2979 | C26H45NO7S | + | HMDB0000036 | ↑ | ↓ | ↓ | ↓ | ↓ |
| LysoPC (22:6) | 26.62 | 567.3310 | C30H50NO7P | + | HMDB0010404 | ↓ | ↑ | ↑ | ↑ | ↑ |
| PC (18:1/18:1) | 28.89 | 785.5907 | C44H84NO8P | + | HMDB0000593 | ↑ |  | ↓ |  |  |
| 7α-Hydroxy-3-oxo-4-cholestenoate | 25.58 | 430.3078 | C27H42O4 | + | HMDB0012458 | ↑ |  |  |  |  |
| L-Methionine | 1.81 | 149.0510 | C5H11NO2S | + | HMDB0000696 | ↑ | ↓ | ↓ |  |  |
| Docosahexaenoic acid | 28.12 | 328.2395 | C22 H32 O2 | + | HMDB0002183 | ↓ |  |  |  |  |
| LysoPC (22:5/0:0) | 27.03 | 569.3468 | C30H52NO7P | + | HMDB0010403 | ↓ |  |  |  |  |
| LysoPE (24:0/0:0) | 26.37 | 565.3122 | C29H60NO7P | + | HMDB0011527 | ↓ |  |  |  |  |
| LysoPC (14:0/0:0) | 25.70 | 467.2999 | C22H46NO7P | + | HMDB0010379 | ↓ |  | ↑ | ↑ | ↑ |
| Chenodeoxyglycocholic acid | 23.69 | 449.31329 | C26H43NO5 |  | HMDB0006898 | ↑ |  | ↑ |  |  |
| 3-Oxo-4,6-choladienoic acid | 21.94 | 370.2505 | C24H34O3 | + | HMDB0000476 | ↑ |  |  |  |  |
| Arachidonic acid | 28.20 | 304.2396 | C20H32O2 | + | HMDB0001043 | ↓ | ↑ | ↑ |  |  |
| LysoPA (19:0/0:0) | 25.58 | 452.2896 | C22H45O7P | + | HMDB0114746 | ↑ |  |  |  |  |
| 5-Hydroxyindole-3-acetic acid | 3.65 | 191.0582 | C10H9NO3 | + | HMDB0000763 | ↓ | ↑ |  |  |  |
| LysoPC (15:0/0:0) | 27.88 | 481.31600 | C23H48NO7P | + | HMDB0010381 | ↓ | ↑ |  | ↑ | ↑ |
| PC (20:1/14:0) | 28.28 | 759.5748 | C42H82NO8P | + | HMDB0008295 | ↑ |  |  |  |  |
| LysoPC (18:1/0:0) | 27.15 | 521.3472 | C26H52NO7P | + | HMDB0002815 | ↓ | ↑ | ↑ | ↑ | ↑ |
| LysoPC (17:0/0:0) | 27.62 | 509.3471 | C25H52NO7P | + | HMDB0012108 | ↓ |  |  |  | ↑ |
| L-Kynurenine | 3.65 | 208.0849 | C10H12N2O3 | + | HMDB0000684 | ↓ |  |  |  |  |
| Dihydrouracil | 8.98 | 114.0470 | C4H6N2O2 | + | HMDB0000076 | ↓ | ↑ |  |  |  |
| Methyl indole-3-acetate | 16.16 | 189.0789 | C11H11NO2 | + | HMDB0029738 | ↑ | ↓ | ↓ |  |  |
| Phytosphingosine | 20.63 | 317.2928 | C18H39NO3 | + | HMDB0004610 | ↑ | ↓ | ↓ |  |  |
| LysoPE (18:2/0:0) | 26.74 | 477.2845 | C23H44NO7P | + | HMDB0011507 | ↑ | ↓ |  |  | ↓ |
| LysoPC (18:2/0:0) | 26.36 | 519.3314 | C26H50NO7P | + | HMDB0010386 | ↓ | ↑ | ↑ | ↑ | ↑ |
| LysoPC (18:0/0:0) | 28.06 | 523.3629 | C26H54NO7P | + | HMDB0010384 | ↓ |  |  | ↑ | ↑ |
| LysoPC (20:3/0:0) | 28.06 | 545.3439 | C28H52NO7P | + | HMDB0010393 | ↓ |  |  | ↑ | ↑ |
| Methyl acetoacetate | 23.67 | 408.2872 | C24H40O5 | － | HMDB0000619 | ↑ |  | ↓ |  |  |
| Deoxycholic acid | 25.22 | 392.2923 | C24H40O4 | － | HMDB0000626 | ↑ |  | ↓ |  | ↓ |
| LysoPC (20:5/0:0) | 26.80 | 541.3380 | C28H48NO7P | － | HMDB0010397 | ↓ | ↑ | ↑ |  |  |
| 3-Oxohexanoic acid | 9.16 | 130.0618 | C6H10O3 | － | HMDB0010717 | ↓ |  |  |  |  |
| 3-Hydroxymethylglutaric acid | 1.41 | 162.0519 | C6H10O5 | － | HMDB0000355 | ↑ |  |  |  |  |
| 4-vinylphenol sulfate | 14.26 | 200.0136 | C8H8O4S | － | HMDB0062775 | ↑ | ↓ |  |  |  |
| 2-Methylacetoacetic acid | 3.44 | 116.0461 | C5H8O3 | － | HMDB0003771 | ↓ | ↑ |  |  | ↑ |
| L-Ascorbic acid 2-sulfate | 1.27 | 255.9885 | C6H8O9S | － | HMDB0060649 | ↓ |  |  |  |  |
| Indole-3-acetylglycine | 13.62 | 232.0841 | C12H12N2O3 | － | HMDB0240661 | ↓ |  |  |  |  |
| Dihydroxyfumaric acid | 1.66 | 147.9891 | C4H6O6 | － | HMDB0002050 | ↑ | ↓ |  |  |  |

Note: ↑ indicates a significant increase compared to the MOD group; ↓ indicates a significant decrease compared to the MOD group.
